# Dynamic Co-evolution and Interaction of Avian Leukosis Virus Genetic Variants and Host Immune Responses

**DOI:** 10.3389/fmicb.2017.01168

**Published:** 2017-06-26

**Authors:** Xuan Dong, Fanfeng Meng, Tao Hu, Sidi Ju, Yang Li, Peng Sun, Yixin Wang, Wenqing Chen, Fushou Zhang, Hongqin Su, Sifei Li, He Cui, Junxia Chen, Shuzhen Xu, Lichun Fang, Huaibiao Luan, Zhenjie Zhang, Shuang Chang, Jianliang Li, Lei Wang, Peng Zhao, Weifeng Shi, Zhizhong Cui

**Affiliations:** ^1^College of Veterinary Medicine, Shandong Agricultural UniversityTaian, China; ^2^Institute of Pathogen Biology, Taishan Medical CollegeTaian, China

**Keywords:** avian leukosis virus subgroup J, genetic variants, immune selective pressure, co-evolution, high-throughput sequencing

## Abstract

Subgroup J avian leukosis virus (ALV-J), a typical retrovirus, is characterized of existence of a cloud of diverse variants and considerable genetic diversity. Previous studies describing the evolutionary dynamics of ALV-J genetic variants mainly focused on the early infection period or few randomly selected clones. Here, we inoculated 30 specific-pathogen-free chickens with the same founder ALV-J stock of known genetic background. Six (three antibody positive and three antibody negative) chickens were selected among 15 chickens with viremia. Viruses were serially isolated in 36 weeks and then sequenced using MiSeq high-throughput sequencing platform. This produced the largest ALV-J dataset to date, composed of more than three million clean reads. Our results showed that host humoral immunity could greatly enhance the genetic diversity of ALV-J genetic variants. In particular, selection pressures promoted a dynamic proportional changes in ALV-J genetic variants frequency. Cross-neutralization experiment showed that along with the change of the dominant variant, the antibody titers specific to infectious clones corresponding to the most dominant variants in weeks 12 and 28 have also changed significantly in sera collected in weeks 16 and 32. In contrast, no shift of dominant variant was observed in antibody-negative chickens. Moreover, we identified a novel hypervariable region in the gp85 gene. Our study reveals the interaction between ALV-J and the host, which could facilitate the development of vaccines and antiviral drugs.

## Introduction

As RNA viruses, retroviruses—such as human immunodeficiency virus (HIV), simian immunodeficiency virus (SIV), and avian leukosis virus (ALV) replicate with extremely high mutation rates due to poor fidelity during reverse transcription. Retroviruses therefore exhibit great genetic diversity (Novella et al., 2014), which allows a viral population to rapidly adapt to various changing environments and evolve resistance to host immune responses and antiviral drugs (Moore et al., 2002; Arias et al., 2014). These viral variants jointly determine the biological characteristics of the population by means of interaction (Lauring and Andino, 2010). There have been numerous studies describing genetic variants in different human and animal RNA viruses, e.g., HIV (Bhattacharya et al., 2007; Keele et al., 2008; Kawashima et al., 2009; McMichael et al., 2010), hepatitis C virus (Dowd et al., 2009), and ALV (Wang and Cui, 2006). However, in these studies, only a limited number of selected clones of polymerase chain reaction (PCR) products from the samples were sequenced, which precluded a thorough understanding of the evolutionary dynamics and population genetics of the viruses. In addition, most previous studies used experimental materials obtained from clinical patients of an unknown background, which might have complicated effect on the results. Many predictive models of viral genetic variants have also provided insight into the association between viral variants and the progression of diseases. However, in some cases, these predictions contrasted with the traditional views of viral behavior and evolution (Lauring and Andino, 2010).

The deep sequencing technique can produce extreme large amounts of sequence data, covering genomic variants of even very low frequencies. The use of deep sequencing has facilitated the understanding of the diversity and evolutionary dynamics of the genetic variants under selective pressure from immune responses and antiviral drugs in various human viruses, especially HIV (Henn et al., 2012; Bansode et al., 2013; Recordon-Pinson et al., 2013).

Aside from being a cause of mortality in poultry (Payne and Nair, 2012), avian retroviruses, generally referred to as ALV, have served as important models promoting the understanding of retrovirology (Weiss and Vogt, 2011). Subgroup J ALV (ALV-J) was first isolated in 1988 from meat-type chickens in the United Kingdom (Payne et al., 1991), and it then spread throughout the world (Gao et al., 2010). ALV-J is easier to mutate than other subgroups of ALV (Payne and Nair, 2012), and is more pathogenic to white meat-type chickens but less pathogenic to layers of different genetic background. Although eradication programs have been successfully conducted in white meat-type chickens, ALV-J has spread to egg-type chickens and many Chinese local breeds of chickens, causing significant economic losses in China over the past 10 years (Sun and Cui, 2007; Gao et al., 2010).

ALV-J exhibits considerable genetic diversity resulting from the high mutation rate of reverse transcriptase, genomic recombination, and selective pressure from antiretroviral therapies and cell-mediated immune responses (Silva et al., 2000). Interestingly, it has been shown that ALV-infected chickens can have four different disease courses depending on their viremia status (V+ or V-) and antibody responses (Ab+ or Ab-) (Payne and Nair, 2012). Among the proteins encoded by ALV, the envelope glycoprotein can interact with receptors on the host cells and determine the subgroup of the viruses. The envelope gene (env) of ALV encodes two proteins, gp85 and gp37. The gp85 protein is located on the surface of the virion and is the major protein for subgrouping ALV.

Pandiri et al. (2010) demonstrated that the emergence of antibody-escape variant in meat-type chickens contributed to ALV-J persistence. Wang and Cui (2006) studied the diversity of ALV-J variants by sequencing one randomly selected clone during a series of passages in cell culture under selective pressure of specific antibodies; they found three hypervariable regions in the gp85 gene. Recently, Meng et al. (2016b) found that there were significant differences in dominant variants and their evolutionary dynamics of ALV-J in chickens and cell cultures.

In the present study, we selected specific-pathogen-free (SPF) chickens as experimental animals and inoculated them with the same founder ALV-J stock. These chickens had viremia, but some of them were antibody-positive, whereas others were antibody-negative. The viruses were serially isolated during a 36-week period and then sequenced using the MiSeq Illumina sequencing platform, producing the largest ALV-J dataset to date. Our study demonstrates the dynamic evolution of ALV-J genetic variants and sheds light on the interaction between ALV-J and its host.

## Materials and Methods

### Virus Strain

The ALV-J field strain NX0101 (GenBank accession number DQ115805) was isolated from a parental chicken breeder farm in the Ningxia Hui Autonomous Region in 2001 (Cui et al., 2003). The infectious clone rNX0101 was constructed, amplified, titered (Zhang et al., 2005), and passaged for 15 generations in DF1 cells, with a median tissue culture infective dose (TCID_50_) of 10^4.5^ per 0.1 ml of DF1 cell culture supernatant.

### Experimental Chickens

One-day-old SPF chickens were purchased from SPAFAS Co. (Jinan, China; a joint venture with Charles River Laboratory, Wilmington, MA, United States). Thirty SPF chickens were inoculated before 1 day of age with 2000 TCID_50_ per bird. Venous blood and anticoagulant blood were collected at 2, 4, 8, 12, 16, 20, 24, 28, 32, and 36 weeks. The experiment was carried out in isolators receiving filtered negative-pressure air.

### Virus Isolation and Virus Antibody Test

Plasma and venous blood samples collected at each sampling time-point were tested for viremia and ALV-J antibody. Blood samples were centrifuged at 2000 rpm for 3 min, and the plasma was used to inoculate a monolayer of DF1 cells, which are resistant to ALV subgroup E (American Type Culture Collection, Manassas, VA, United States). The uninfected DF1 cells were used as a negative control. After inoculation, the DF1 cells were incubated at 37°C for 2 h. Then, the culture medium was discarded and replaced with fresh Dulbecco’s modified Eagle’s medium (DMEM) supplemented with 2% fetal bovine serum (FBS) and further cultured for 7–10 days at 37°C. Approximately 200 μl of the cell lysate was used to assay the p27 group-specific antigen using an Avian Leukosis Virus Antigen Test Kit (IDEXX, United States), according to the manufacturer’s instructions. Venous blood samples collected at each sampling time-point were tested for ALV-J antibodies by using a Subgroup J Avian Leukosis Virus Antibody Test Kit (IDEXX, United States), in accordance with the manufacturer’s instructions.

### Primers

Two pairs of primers (gp85-A-F: 5′-GGCATTCCACAGTATCCTC-3′, gp85-A-R: 5′-CGTCCATGATTGGTTGACA-3′; gp85-B-F:5′-GTCCAATAAACGTAGAGAG-3′, gp85-B-R: 5′-GCCCTGTCCCCACAAATCA-3′) were designed to amplify the two highly variable regions of the gp85 gene, with gp85-A including the vr2 and hr1 regions and gp85-B including the hr2 and vr3 regions. Each sample was amplified using a forward primer with a six-digit error-correcting barcode as described earlier (Hamady et al., 2008). In addition, a 2-bp GT linker was added between the barcode and the 5′ end of the F primer to avoid a potential match between the barcode and the target sequences.

### RNA Preparation, gp85 Cloning, and High-Throughput Sequencing

For each sample, 50 μl plasma was used to extract viral RNA using the MagMAX-96 viral RNA isolation kit (Ambion, Austin, TX, United States) according to the manufacturer’s instructions. The cDNA was generated with the reverse primers using the TaKaRa RNA PCR Kit (AMV) Ver.3.0 (Takara Bio, Shiga, Japan). The PCR conditions were as follows: denaturation at 94°C for 2 min; 32 cycles of denaturation at 94°C for 15 s, annealing at 58°C for 30 s, and extension at 68°C for 30 s; and a final elongation step at 68°C for 10 min. The PCR products were run on 1% agarose gels and extracted using the QIAquick gel extraction kit (Qiagen, Hilden, Germany). The products were quantified using a spectrophotometer (NanoDropND-1000, Thermo Fisher Scientific, Waltham, MA, United States).

The barcode-tagged products of the same fragment were pooled and purified using the QIAquick PCR Purification Kit (Qiagen, Hilden, Germany). The DNA was end-repaired, A-tailed, and PE-adapter ligated. After ligation of the adapters, each sample was purified using the QIAquick Gel Extraction Kit (Qiagen, Hilden, Germany) and sequenced using the PE250 strategy on an Illumina MiSeq according to the manufacturer’s instructions. A base-calling pipeline (Sequencing Control Software, SCS; Illumina) was used to process the raw fluorescent images and the called sequences.

### Data Filtration and Assembly

The raw data were pre-processed following in-house procedures to remove reads of low quality. If the two paired-end reads overlapped, the consensus sequence was generated using COPE, V1.2.1 (Liu et al., 2012). Reads were filtered using instrument quality scores and aligned to the reference ALV-J sequence (DQ115805) using a codon-aware version of the Smith–Waterman algorithm that can correct homopolymer errors by considering both nucleotide and amino acid homology. Multiple sequence alignment was performed using Muscle (Edgar, 2004) and then manually adjusted. All the clean data obtained have been submitted to the Sequence Read Archive (SRA) under accession no. PRJNA385044.

### Shannon Entropy and Positive Selection Analysis

To minimize potential sampling bias and reduce the computation load, we performed a bootstrapping strategy according to the numbers of clean reads (Supplementary Table S1), which has been previously applied in previous studies (Domingo et al., 2004; Meng et al., 2016a). For each re-sampling, the Shannon entropy was calculated as previously described (Meng et al., 2016a). Phylogenetic trees were constructed using RaxML (Stamatakis et al., 2005) and used as input trees in HyPhy (Pond et al., 2005). Global selection pressure values (ω) were estimated using HyPhy. Mean values were estimated for the bootstrapped datasets. Site-by-site positive selection was also performed using HyPhy for each bootstrap replicate. Two methods were applied, the two rate fixed-effects likelihood (FEL) and the Mixed Effects Model of Evolution (MEME) (Kosakovsky Pond and Frost, 2005; Murrell et al., 2012). Maximum-likelihood trees obtained from the previous step were also used as input trees. *P*-values of <0.05 were considered significant.

### Microneutralization

Two full-length ALV-J infectious clones with the gp85 genes from the most dominant variants of the former and the later periods in the plasma were generated using reverse genetics as previously described (Zhang et al., 2005), named as rNX0101-E (possessing the gp85 gene from week 12 of chicken 1) and rNX0101-L (possessing the gp85 gene from week 28 of chicken 1), respectively. In brief, 4 μg purified plasmids rNX0101-E and rNX0101-L were transfected into DF1 cells in monolayer using Lipofectamine 2000 (Invitrogen, Carlsbad, CA, United States). The culture supernatant containing the virus stocks was harvested for 7 days and quantified TCID_50_.

Cross-neutralization assays were performed to detect the dynamic change of the antibodies in the former (16 weeks of chicken 1) and later periods (32 weeks of chicken 1). Antibodies were titered for neutralizing activity against each viruses as follows. Two-fold serial dilutions of each Ab were incubated with 100 TCID_50_ of virus in PBS at 4°C for 1 h. DF1 cells monolayers in 96-well plates were washed once with PBS and inoculated with virus-antibody mixtures. Following incubation for 2 h at 37°C in 5% CO_2_, the inoculum was removed and monolayers were again washed once with PBS. DMEM supplemented with 2% FBS was added and cells were incubated for 7 days at 37°C. The presence of virus in cell culture supernate was assessed by ELISA assays using Avian Leukosis Virus Antigen Test Kit (IDEXX, United States).

### Ethics Statement

The experiment was carried out in isolators receiving filtered negative-pressure air in the animal farm of Shandong Agricultural University. This study was reviewed and approved by the Shandong Agricultural University Animal Care and Use Committee (ID SDAUA-2014-007) and performed in accordance with the “Guidelines for Experimental Animals” of the Ministry of Science and Technology (Beijing, China). Different groups were separated and individually identifiable with wing bands. Any bird deemed to have reached the humane endpoint was culled.

## Results

### Selection of Experimental Animals

**FIGURE 1:**
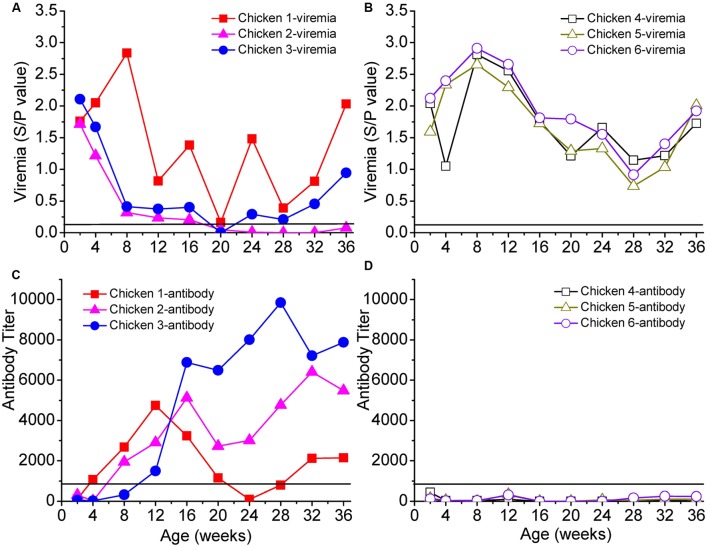
Viremia and neutralizing antibodies of six selected chickens. Panel **(A)** shows the persistent viremia of chickens 1, 2, and 3, and panel **(B)** shows the persistent viremia of chickens 4, 5, and 6. Panels **(C,D)** show the antibody responses of chickens 1–3 and 4–6, respectively. The *x*-axis represents the ages of the chickens in weeks. Solid lines in each panel represent the cut-off values.

We selected six chickens with persistent viremia, of which three were antibody-positive (chickens 1–3) and three were antibody-negative (chickens 4–6) (**Figure 1**). Chicken 1 was positive for the ALV antibody during weeks 4–20; the antibody then decreased and disappeared but reappeared at the 32nd week. Chickens 2 and 3 were positive for the ALV antibody after the 8th week.

### Deep Sequencing and Data Filtering

Raw reads were filtered, aligned, trimmed, and translated using pre-specified criteria, so that all sequences included the complete hypervariable and variable regions of the gp85 gene. For hypervariable region A (gp85-A, including the vr2 and hr1 regions), the alignment was 327 bp in length. The total number of clean reads for gp85-A was approximately 1.66 million, with a mean value of ∼30,000 reads per chicken per sampling time-point (Supplementary Table S2). For hypervariable region B (gp85-B, including the vr3 and hr2 regions), the alignment was 270 bp in length. After removing reads of low quality, this left ∼1.41 million clean reads, with a mean value of ∼25,700 reads per chicken per sampling time-point (Supplementary Table S2).

### Dynamic Evolution of gp85-A and gp85-B Genetic Variants in Antibody-Positive and Antibody-Negative Chickens

**FIGURE 2:**
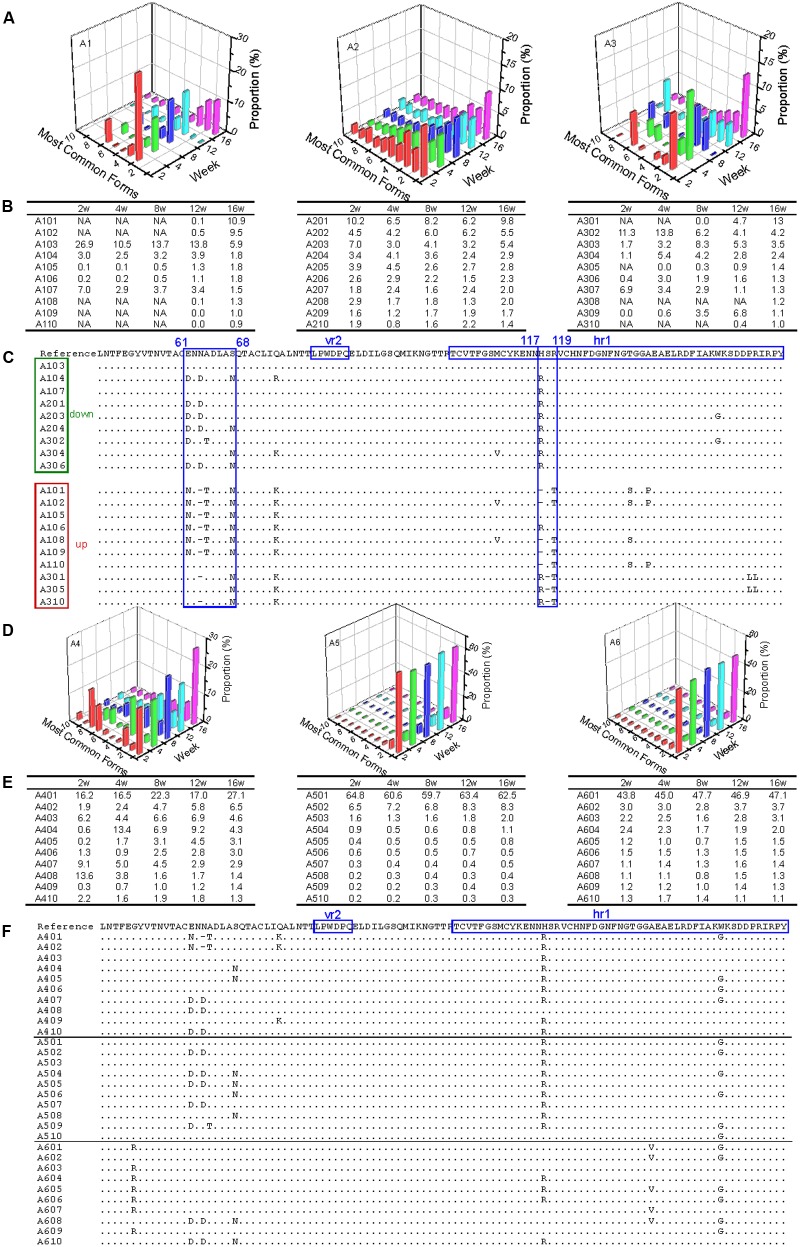
Evolutionary dynamics of gp85-A (vr2 and hr1 regions) genetic variants in antibody-positive and antibody-negative chickens during the first 16 weeks. In this figure, the variants are ordered according to their ranking at week 16. Chickens 1–3 were antibody-positive, and 4–6 were antibody-negative, as in **Figure 1**. In panels **(B,C,E,F)**, the variants are named using the same strategy. For example, in A101, “A” refers to region A of the gp85 gene (gp85-A), the first number “1” means chicken 1, and “01” represents the top 1 variant at week 16; therefore, A101 represents the top 1 gp85-A variant of chicken 1 at week 16. In panels **(B,E)**, NA means that no data are available. Panels **(A,D)** show the proportions of the top 10 variants of chickens 1–3 and 4–6 during the 16 weeks. Panels **(C,F)** show the amino acid substitutions of the variants of chickens 1–3 and 4–6, respectively. In the two panels, the dominant variant when inoculated is used as the reference, and the amino acid positions are numbered from the first codon of the mature gp85 protein. The two previously identified variable regions, vr2 and hr1, are highlighted. In panel **(C)**, the representative variants are classified into two groups, down and up, in which the proportion of the variants decreases or increases with time, respectively. The most dominant variant in the inoculated stock is used as the reference, and the amino acid positions are numbered from the first codon of the mature gp85 protein.

In the three antibody-positive chickens (chickens 1–3), there was no distinct dominant variant for gp85-A, and the proportion of the first-ranked variant was relatively small, generally <15%. In addition, there was a dynamic change of the top 10 variants. For example, some variants emerged, and their percentages gradually increased, such as A101, A102, and A301. In contrast, the percentages of some variants gradually decreased, e.g., A103 and A302 (**Figures 2A,B**). Non-synonymous substitutions and deletions were found in 14 amino acid sites, half of which fell within the hr1 region (Wang and Cui, 2006) (**Figure 2C**). However, no amino acid mutations were found in the vr2 region. In particular, there were four amino acid substitutions falling within positions 61–68, suggesting a novel hypervariable region, which had not been previously described.

In contrast, in the three chickens that were antibody-negative but had persistent viremia (chickens 4–6), there were dominant variants (A401 for chicken 1, A501 for chicken 5, and A601 for chicken 6). The percentages of the dominant variants were relatively large and changed slightly during weeks 2–16, such as 60% for A501 and ∼45% for A601 (**Figures 2D,E**). It was also notable that these dominant variants were not 100% identical and they emerged very quickly (before week 2). Nine amino acid substitutions were observed, with three falling within the hr1 region (**Figure 2F**). Interestingly, the remaining six amino acid substitutions did not fall within the vr2 region either.

Results from the gp85-B analysis revealed a similar pattern of dynamic change (Supplementary Figure S1). On the one hand, a dominant variant (>50%) existed in the antibody-negative chickens but not in the antibody-positive ones. In addition, there was a dynamic and complicated change of the ALV variants in the antibody-positive chickens, which was not observed in the antibody-negative chickens. More amino acid positions experienced non-synonymous substitutions in the antibody-positive than in the antibody-negative chickens, and most of them were located within the known variable regions (hr2 and vr3), particularly at positions 189–192.

### Dynamic Co-evolution of ALV and Chickens

**FIGURE 3:**
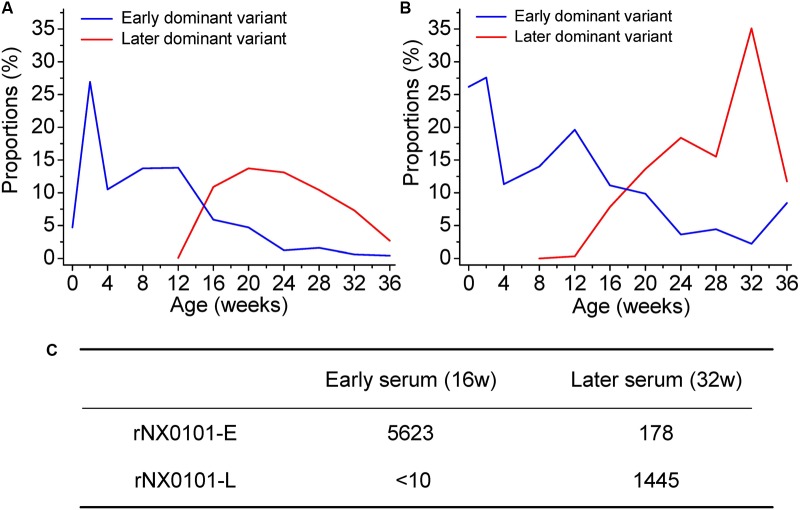
Dynamic co-evolution of ALV and host humoral immunity in chicken 1. **(A)** The shift of dominant variant in region A of the gp85 gene (gp85-A). **(B)** The shift of dominant variant in region B of the gp85 gene (gp85-B). The blue line indicates the early dominant variant, while the red line indicates the later dominant variant. **(C)** The result of cross-neutralization between the early/later sera and two infectious clone viruses representing the early/later most dominant variants. rNX0101-E means the early dominant variant possessing the gp85 gene of the most dominant variant in plasma at week 12 of chicken 1. rNX0101-L means the later dominant variant possessing the gp85 gene of the most dominant variant in plasma at week 28 of chicken 1.

Our results have revealed the dynamic change of the genetic variants of ALV-J under host humoral immunity (**Figures 3A,B**). To further study the dynamic co-evolution of ALV-J genetic variants and chickens, we performed cross neutralization assays to display the dynamic change of antibodies along with the change of ALV-J variants. Chicken 1 was taken as an example. First, in our experiment, the antibody level of chicken 1 was the highest in week 12, however, it gradually decreased to below the detection limit in week 24. After that, it gradually increased and reached a platform from week 32. This indicated that there might be a shift of antibodies in chicken 1. Second, the proportion of the dominant ALV variant also gradually decreased in chicken 1, whereas a novel variant emerged from week 12 and became one of the dominant variants (**Figures 3A,B**). This was consistent with the change of the antibody level of chicken 1.

To reveal the dynamic change of the antibodies in weeks 16 and 32 in chicken 1, both homologous neutralization and cross-neutralization experiments were performed. Our results showed that serum from week 16 neutralized rNX0101-E with a titer of 5623, whereas it was 178 for serum from week 32. On the contrary, serum from week 32 neutralized rNX0101-L with a titer of 1445, whereas it was <10 for serum from week 16 (**Figure 3C**). This suggested that the antibodies from the former and later periods were different.

### Genetic Diversity of the ALV-J Variants

**FIGURE 4:**
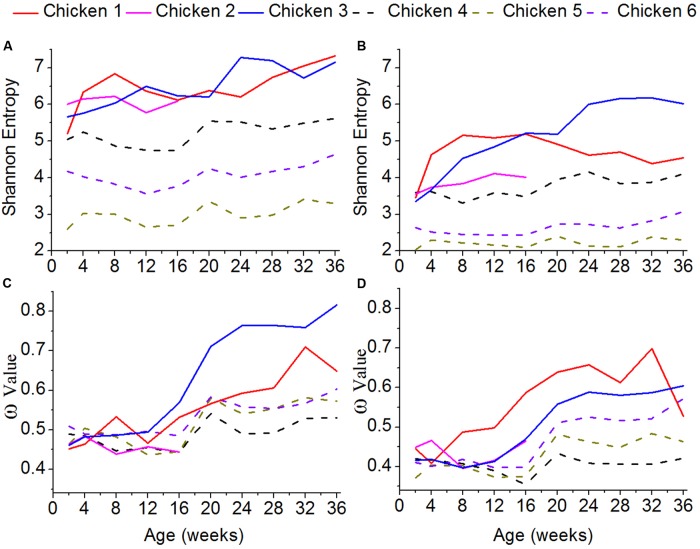
Shannon entropy and global selection pressures (ω) estimated using quasispecies at different time-points. **(A)** The shannon entropy values calculated for gp85-A. **(B)** The values for gp85-B. **(C)** The global selection pressures (ω) calculated using the quasispecies of chickens 1–3 for gp85-A. **(D)** The global selection pressures (ω) calculated using chickens 4–6 for gp85-B.

To assess the genetic diversity of the ALV variants, the Shannon entropy was estimated (**Figures 4A,B**). The higher the Shannon entropy values are, the higher genetic diversity ALV-J has. For both of gp85-A and gp85-B, the Shannon entropy values of the antibody-positive chickens (chickens 1–3) were higher than those of the antibody-negative ones (chickens 4–6), suggesting that antibody responses might be associated with and most likely enhance the genetic diversity of ALV. In addition, for both gene regions, the Shannon entropy values gradually increased. It is worth noting that the Shannon entropy of gp85-A was higher than that of gp85-B.

### Global Selection Pressures

To roughly quantify the immunological pressures that ALV-J underwent, we calculated the global selection pressure values (ω) using HyPhy (**Figures 4C,D**). Our results showed that the changes of the ω values were consistent with those of the Shannon entropy. Specifically, the ω values of the antibody-positive chickens (chickens 1–3) were higher than those of chickens 4–6, lacking antibody responses. Moreover, the ω values gradually increased for both gp85-A and gp85-B during our experimental stage.

### Sequence Similarity

**FIGURE 5:**
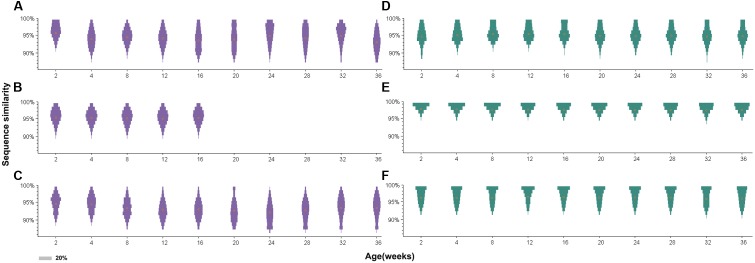
Distribution of sequence similarity of the gp85-A quasispecies during a 36-week period. **(A–C)** The sequence similarity of chickens 1–3. **(D–F)** The sequence similarity of chickens 4–6. The *x*-axis of each panel represents different sampling time-points. The *y*-axis represents sequence similarity. The orange circle represents the mean value of sequence similarity. The width of the bars represents the proportion of this sequence similarity value, and the legend is shown in the lower left corner.

Further analysis of sequence similarity in the clean reads revealed similar results (**Figure 5**). In the antibody-positive chickens, the reads of gp85-A had low sequence similarities, with the mean values usually <95%. In addition, only few reads were 100% identical. However, in chickens without antibody responses, a large fraction of the reads were identical, and the average sequence similarities were greater than 95%. The gp85-B analysis also showed that the reads from the antibody-negative chickens generally had higher sequence similarities than those from the antibody-positive chickens (Supplementary Figure S2).

Furthermore, for the antibody-positive chickens (chickens 1–3), the range of sequence similarities became wider over time; in contrast, it just slightly changed for the antibody-negative chickens (chickens 4–6). For example, the sequence similarities of gp85-A of the top 10 variants for chicken 2 at week 2 ranged from 95.4 to 99.1%, but the range became 91.6–99.1% at week 36. Similarly, for chicken 3, the sequence similarities of the top 10 variants ranged from 92.6 to 99.1% at week 2 and from 88.9 to 99.1% at week 28. Conversely, for chicken 6, the sequence similarity range of the top 10 variants did not change during the 36 weeks, remaining between 95.4 and 99.1%.

### Site-by-Site Positive Selection Analysis

**FIGURE 6:**
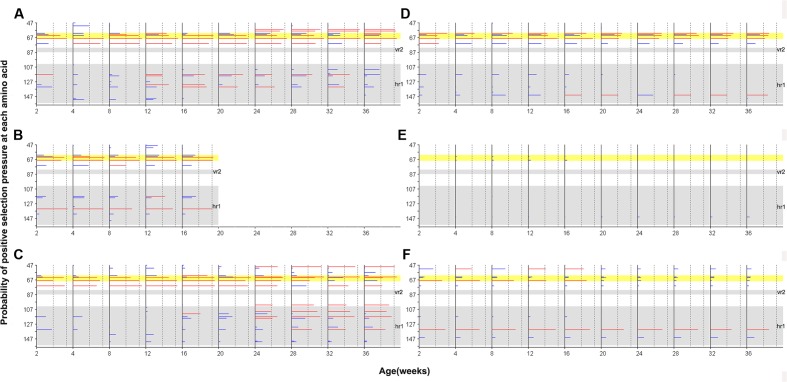
Site-by-site positive selection of the gp85-A gene based on the two rate FEL method. **(A–C)** The value of site-by-site positive selection in chickens 1–3. **(D–F)** The value of site-by-site positive selection in chickens 4–6. The *x*-axis of each panel represents different sampling time-points. The *y*-axis represents the amino acid positions of the gp85 protein. The horizontal lines in each panel represent the positively selected sites, and the length of the lines represents the proportion of the identified positively selected sites in the bootstrap re-sampling replicates. Red lines indicate that these amino acid sites are positively selected in at least 50% of the bootstrap re-sampling replicates, and blue lines indicate positive selection of less than 50%. The two vertical dashed lines represent the 50% and 90% thresholds, respectively. The two previously described hypervariable regions, vr2 and hr1, are shown using a gray background. Positions 61–68, a novel hypervariable region, are highlighted using a yellow background.

Site-by-site positive selection analysis using the two rate FEL method showed that a total of 17 amino acid sites of gp85-A were under positive selection; these sites were found to be positively selected in more than 50% of the bootstrap replicates (**Figure 6** and Supplementary Tables S3, S4). Fifteen sites were found in the antibody-positive chickens (chickens 1–3), whereas only seven sites were found in the antibody-negative ones (chickens 4–6) and no sites were under positive selection in chicken 5. The identified positively selected sites were generally consistent with the mutation sites shown in **Figure 2**. Among the 17 sites, six (110, 113, 117, 119, 131, and 134) fell within the hr1 region, however, no positively selected sites were found in the vr2 region. In particular, four adjacent sites (61, 63, 64, and 68) were positively selected, forming a novel variable region described here for the first time (**Figure 6**). Results obtained using the MEME method were broadly consistent (Supplementary Figure S3).

Furthermore, results obtained using gp85-B were in accordance with those of gp85-A. Nine positively selected amino acid sites were identified in chickens with positive antibody responses, with three in the hr2 region and three in the vr3 region. These regions were previously shown to undergo multiple amino acid substitutions (Supplementary Figure S4 and Tables S5, S6). Only one site, 190, was likely under positive selection in chicken 4. No positively selected sites were identified in chickens 5 and 6, whose antibody responses were negative. Additional results, obtained using the MEME method for gp85-B, are shown in Supplementary Figure S5.

**FIGURE 7:**
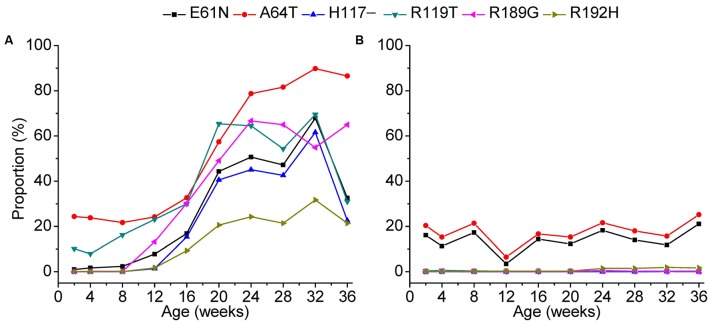
Fixation of amino acids under positive selection during a 36-week period. The fixation of the amino acids under positive selection in chickens 1–3 was displayed in panel **(A)** and that of chickens 4–6 in panel **(B)**. The *x*-axis of each panel represents different sampling time-points. The *y*-axis represents the mean proportion of the variants with the amino acid substitution in the three experimental chickens.

Our data further revealed that site-by-site positive selection led to the fixation of specific amino acid substitutions in the antibody-positive chickens (**Figure 7**). Positions 61, 64, 117, and 119 of gp85-A and 189 and 192 of gp85-B were identified as positively selected. Accordingly, amino acid substitutions at these positions displayed a different amino acid substitution pattern between antibody-positive and antibody-negative chickens. In chickens with positive antibody responses, the proportions of E61K, A64T, H117, R119T, R189G, and R192H increased gradually at different sampling time-points, suggesting a dynamic fixation process (**Figure 7**). However, in chickens without antibody responses, the proportions of these amino acid substitutions changed slightly, and no amino acid substitutions were fixed.

## Discussion

ALV-J has been found to infect many types of chickens with various genetic backgrounds (Payne and Nair, 2012). Although eradication programs have been successfully conducted in white meat-type chickens, the virus has caused significant economic losses in egg-type chickens and many local breeds of chickens in China during the past 10 years (Sun and Cui, 2007; Gao et al., 2010). However, limited understanding of its complex genetic variations and interaction with the host prevents the development of vaccines and anti-viral drugs. In addition, the background of the hosts from which the viral samples were isolated was unclear. In the present study, we used ALV-J-infected SPF chickens as an animal model to explore the evolutionary dynamics of ALV-J. The use of a uniform biological background could thus avoid the potential influence from the host. In particular, it could provide an insight into how the host humoral immunity influence the ALV-J genetic variations and dynamics.

Numerous studies have revealed the limitations of conventional Sanger sequencing in studying complex viral genetic variants (Holmes, 2007; Haagmans et al., 2009). However, recent advances in the sequencing technology have dramatically reduced the cost, which makes sequencing become a standard technique for surveillance of viral pathogens (Henn et al., 2012). Here, we have used high-throughput sequencing to study ALV-J genetic variants, producing more than three million clean reads in total. To our knowledge, this is the first time that high-throughput sequencing is used to study ALV-J genetic variation under pressures from host humoral immunity, and is the largest ALV-J dataset described.

Our results showed that the three chickens with persistent viremia and positive antibody responses had a higher genetic diversity, indicated by higher Shannon entropy values and lower sequence similarities. Additionally, these chickens seemed to be subject to higher immunological pressures. Therefore, immunological pressure from the host may increase the ALV-J genetic variation, and a dynamic change in the dominant variant was observed under immunological pressures. In particular, cross-neutralization experiments showed that along with the change of the dominant variant, the antibodies produced by chickens have also changed. This revealed the dynamic co-evolution and interaction between ALV-J genetic variants and chickens.

In contrast, chickens with negative antibody responses had lower Shannon entropy values and higher sequence similarities, indicative of lower ALV-J genetic diversity. In this case, replacement of the dominant variant was less likely to occur. Therefore, the antibody response of the host is a major determinant of the complexity and dynamic change of the ALV-J genetic variants, which potentially impacts viral persistence, pathogenicity and the outcome of antiviral therapy. Further, the same technology and similar animal experiment designs could be used to further study the molecular mechanism of ALV-J evolution on its pathogenicity when it spreads among chicken breeds with different genetic backgrounds. It should also be noted that for chickens without antibody, such as chickens 4–6, viremia first increased and then decreased. However, after 28 weeks post infection, viremia increased again, suggesting that unknown pressure(s) other than antibody exist.

A number of hypervariable gene regions have been identified previously (Wang and Cui, 2006; Pandiri et al., 2010; Meng et al., 2016b). We have recently identified a variable region X and the LSD insert in gp85-B in antibody-negative chickens (Meng et al., 2016b). In the present study, our deep sequencing also identified a novel hypervariable gene region at positions 61–68 of the gp85 gene. In this region, the amino acid mutations in the antibody-positive and antibody-negative groups were different, suggesting that antibodies recognizing the novel variable region neutralize ALV infection, and the region is involved in the ALV infection.

## Conclusion

We studied the ALV-J genetic variants isolated from SPF chickens using deep sequencing and presented the largest dataset of ALV-J to date. Our data revealed the dynamic co-evolution and interaction between ALV-J variants and host humoral immunity. Furthermore, we identified a novel hypervariable region in the gp85 gene. To our knowledge, this is the first study providing quantitative experimental data on the evolutionary kinetics of ALV-J under selective pressures from the chicken immune response, using next-generation sequencing. This system may also be used as an animal model to further understand the interaction between retroviruses and their hosts.

## Author Contributions

XD and FM designed and conducted the study, performed most of the experiments, and wrote the manuscript. TH performed the calculation with support from WC, FZ, HS, SL, and HC. SJ, YL, PS, and YW collected samples, prepared RNA, and cloned the gp85 gene. JC, SX, LF, and HL performed the biological experiments. ZZ, SC, JL, LW, and PZ discussed the results. ZC and WS designed the study and wrote the manuscript.

## Conflict of Interest Statement

The authors declare that the research was conducted in the absence of any commercial or financial relationships that could be construed as a potential conflict of interest.
