# Supplementary material for: Dynamic Co-evolution and Interaction of Avian Leukosis Virus Genetic Variants and Host Immune Responses
Source: Front Microbiol. 2017 Jun 26;8:1168. doi: 10.3389/fmicb.2017.01168 (PMC5483431; doi:10.3389/fmicb.2017.01168)
Supplement: Supplementary file 1 [file Data_Sheet_1.pdf]

Dynamic Co-Evolution and Interaction of Avian Leukosis Virus Genetic Variants and Host Humoral Immunity

Xuan Dong[§, 1], Fanfeng Meng[§, 1], Tao Hu[2], Sidi Ju[1], Yang Li[1], Peng Sun[1], Yixin Wang[1], Wenqing Chen[1], Fushou Zhang[1], Hongqin Su[1], Sifei Li[1], He Cui[1], Junxia Chen[1], Shuzhen Xu[1], Lichun Fang[1], Huaibiao Luan[1], Zhenjie Zhang[2], Shuang Chang[1], Jianliang Li[1], Lei Wang[1], Peng Zhao[1], Weifeng Shi[*, 2] and Zhizhong Cui[*, 1]

[1]College of Veterinary Medicine, Shandong Agricultural University, Taian, Shandong, China

[2]Institute of Pathogen Biology, Taishan Medical College, Taian, Shandong, China

[§]These authors contributed equally to this work.

[*]**Corresponding author:** Zhizhong Cui, zzcui@sdau.edu.cn; Weifeng Shi, shiwf@ioz.ac.cn.

A

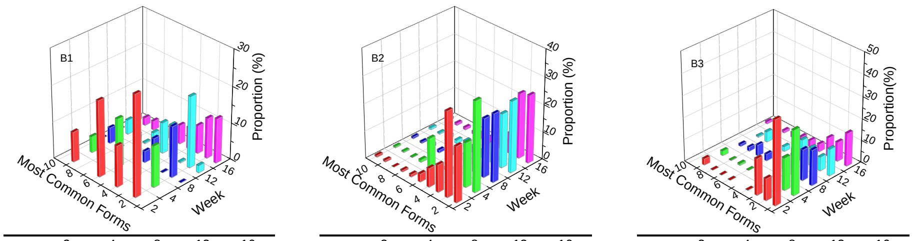

B

	2w	4w	8w	12w	16w
B101	NA	NA	0.0	2.0	12.3
B102	27.6	11.3	14.0	19.6	11.1
B103	NA	NA	0.0	0.3	7.8
B104	11.3	9.9	8.1	9.2	6.0
B105	NA	NA	3.1	8.5	5.2
B106	20.8	13.4	5.7	4.4	3.8
B107	NA	NA	NA	0.3	2.8
B108	NA	NA	0.0	1.2	2.5
B109	8.3	4.5	4.4	4.0	2.1
B110	NA	NA	0.0	0.1	1.2

	2w	4w	8w	12w	16w
B201	20.4	32.9	25.0	26.1	24.9
B202	30.9	16.3	21.6	19.9	23.9
B203	10.1	5.0	9.3	7.5	7.7
B204	6.9	3.0	5.2	3.1	3.5
B205	3.0	1.3	2.7	2.8	2.8
B206	0.9	10.6	3.2	2.7	2.6
B207	0.6	0.9	1.0	1.2	1.2
B208	0.0	0.1	1.3	1.1	0.9
B209	0.7	0.5	0.6	0.5	0.8
B210	0.7	0.5	0.7	0.7	0.7

	2w	4w	8w	12w	16w
B301	38.4	29.6	16.3	12.3	15.2
B302	10.0	14.9	14.2	6.4	8.9
B303	16.7	19.7	12.3	8.4	8.7
B304	0.1	0.4	6.0	9.8	3.4
B305	NA	NA	0.0	0.8	2.9
B306	0.0	0.0	3.1	2.2	2.5
B307	0.1	0.8	5.1	8.3	2.4
B308	0.1	0.2	1.8	4.8	1.0
B309	2.9	2.2	1.0	0.6	1.0
B310	NA	NA	NA	NA	1.0

C

```
                     189  hr2 192                                          vr3
Reference SFSISSRYCGFTSNETRYYRRNRSSWCSSKGGEWSAGYSNGTECSSNTRGCGGNCTAEWNYYAYGFTFGKQPEVLWNNGTAKALPPGIFL
B102      ..........................................................................................
B104      ..........................................K.....T.........................................
B106      ..........................................K..........................GK..I................
B109      .....................................................................GK..I................
B201 down ..........................................K.....T.........................................
B206      ................................................T.........................................
B301      ..........................................................................................
B302      ..........................................K.....T.........................................
B303      .....................................................................GK..I................
B309      ..........................................K...............................................

B101      ...................GG.S...................K.....T.........................................
B103      ...................GGSH...................K.....T.........................................
B105      ...................G..S...................K.....T.........................................
B107      ...................GGSH.........................T.........................................
B108 up   ...................G..S.........................T.........................................
B110      ...................G..S............V......K.....T.........................................
B305      .....R..............GDL.........................T....................GK..I................
B310      .....R..............GDL.N..G..R...........K.....T...................E.....................
```

D

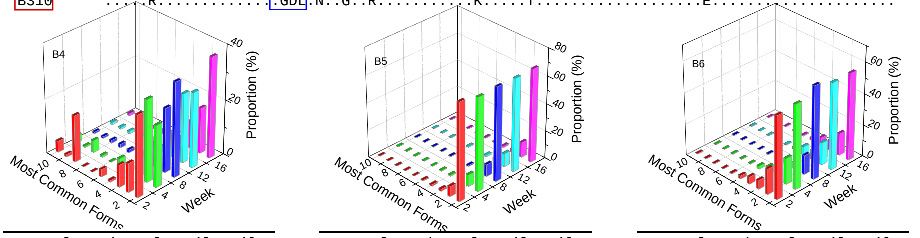

E

	2w	4w	8w	12w	16w
B401	29.6	22.5	34.4	27.4	36.8
B402	10.6	29.9	23.4	25.3	16.7
B403	8.1	8.9	11.0	9.9	10.3
B404	0.6	1.5	1.7	2.8	2.6
B405	2.7	3.2	1.1	2.2	1.6
B406	0.0	0.1	1.0	2.8	1.6
B407	0.1	0.6	1.3	1.5	1.2
B408	17.0	4.4	1.0	0.9	1.1
B409	0.9	0.5	0.6	0.7	1.0
B410	4.1	2.6	0.6	0.8	0.9

	2w	4w	8w	12w	16w
B501	70.9	67.6	68.2	67.7	67.9
B502	7.3	8.5	8.7	9.7	10.9
B503	1.5	1.9	2.0	2.2	2.1
B504	1.1	0.8	0.8	1.2	1
B505	0.6	0.4	0.5	0.7	0.6
B506	0.1	0.1	0.3	0.6	0.3
B507	0.2	0.2	0.2	0.4	0.2
B508	0.1	0.1	0.1	0.2	0.2
B509	0.1	0.1	0.1	0.1	0.1
B510	0.1	0.2	0.1	0.1	0.1

	2w	4w	8w	12w	16w
B601	52.6	53.6	59.2	55.4	55.5
B602	16.2	16.6	12.1	14.2	14
B603	5.6	5.3	4.5	6.3	6.2
B604	4.5	4.6	3.2	5.3	5.0
B605	2.0	1.9	1.7	1.6	1.7
B606	0.4	0.3	0.3	0.4	0.5
B607	0.1	0.1	0.1	0.1	0.4
B608	0.2	0.3	0.1	0.2	0.3
B609	0.2	0.2	0.2	0.1	0.2
B610	0.3	0.2	0.3	0.3	0.2

F

```
                     189  hr2 192                                          vr3
Reference SFSISSRYCGFTSNETRYYRRNRSSWCSSKGGEWSAGYSNGTECSSNTRGCGGNCTAEWNYYAYGFTFGKQPEVLWNNGTAKALPPGIFL
B401      .........................................................................I................
B402      ..........................................K.....T.........................................
B403      ..........................................................................................
B404      ..........................................K.....T........................I................
B405      ..........................................K.....T....................GK..I................
B406      ....................G.....................K.....T.........................................
B407      ................................................T.........................................
B408      ..........................................K..........................GK..I................
B409      ..........................................K..............................I................
B410      .....................................................................GK..I................
B501      .........................................................................I................
B502      .....................................................................GK..I................
B503      ..........................................K.....T.........................................
B504      ..........................................................................................
B505      ..........................................K.....T........................I................
B506      ..........................................K..........................GK..I................
B507      ..........................................K..............................I................
B508      ......................H..................................................I................
B509      ...................G.....................................................I................
B510      .........................................................................I.........P......
B601      .....................................................................GK..I................
B602      ..........................................K...............................................
B603      ..........................................................................................
B604      ..........................................K..........................GK..I................
B605      ..........................................K.....T.........................................
B606      ..........................................K.....T....................GK..I................
B607      .........................................................................I................
B608      R....................................................................GK..I................
B609      .....................................................................GK...................
B610      ................................V....................................GK..I................
```

Fig S1. Evolutionary dynamics of gp85-B (hr2 and vr3 regions) genetic variants in antibody-positive and -negative chickens during the first 16 weeks.

Chickens 1-3 were antibody-positive, and 4-6 were antibody-negative, as in Figure 1. (**A** and **D**) The proportions of the top ten variants of chickens 1-3 and 4-6 at week 16. (**B** and **E**) The dynamic proportional changes in top ten variants at week 16 in chickens 1-3 and 4-6, respectively. NA means no available data. (**C** and **F**) The amino acid substitutions of the variants of chickens 1-3 and 4-6, respectively. In the two panels, the representative variants were classified into two groups, down and up, in which the proportion of the variants decreases or increases over time, respectively. The most dominant variant when inoculated is used as the reference, and the amino acid positions are numbered from the first codon of the mature gp85 protein. The two previously identified variable regions are highlighted. The variants are ordered according to their rankings at week 16. For example, in B101, "B" refers to region B of the gp85 gene (gp85-B), the first number "1" means chicken 1, and "01" represents the top one variants at week 16; therefore, B101 represents the top one gp85-B variant of chicken 1 at week 16.

Figure S2

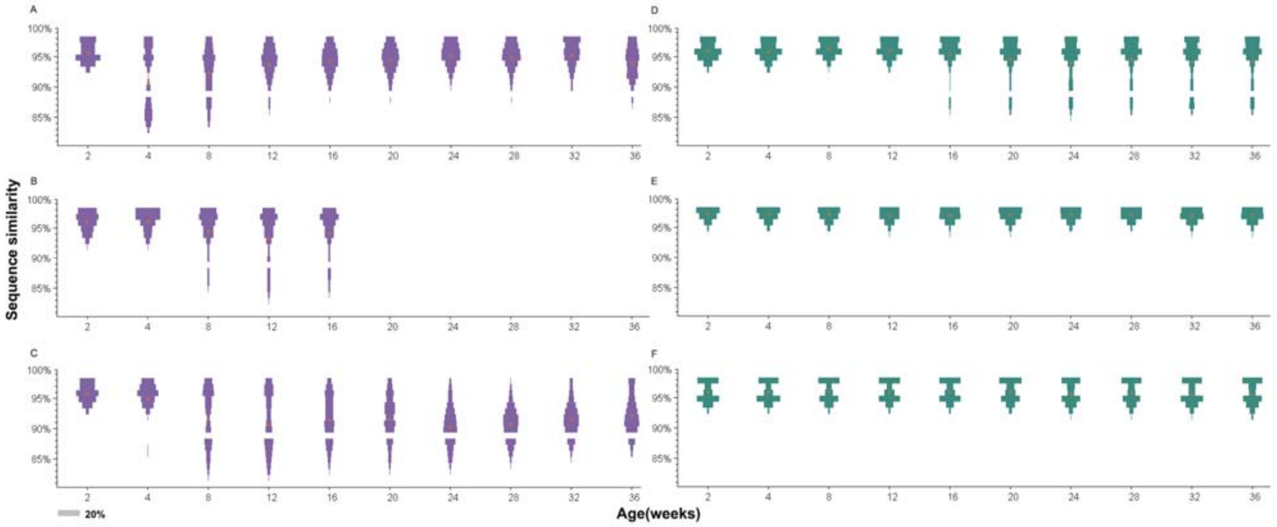

Fig S2. Distribution of sequence similarity of the gp85-B genetic variants during a 36-week period.

(**A**, **B** and **C**) The sequence similarity of chickens 1-3, respectively. (**D**, **E** and **F**) The sequence similarity of chickens 4-6, respectively. The x-axis of each panel represents different sampling time-points. The y-axis represents sequence similarity. The red bar represents 100% identity, and the orange circle represents the mean value of sequence similarity. The width of the grey bar represents the proportion of this sequence similarity value, and the legend is given in the lower left corner.

Figure S3

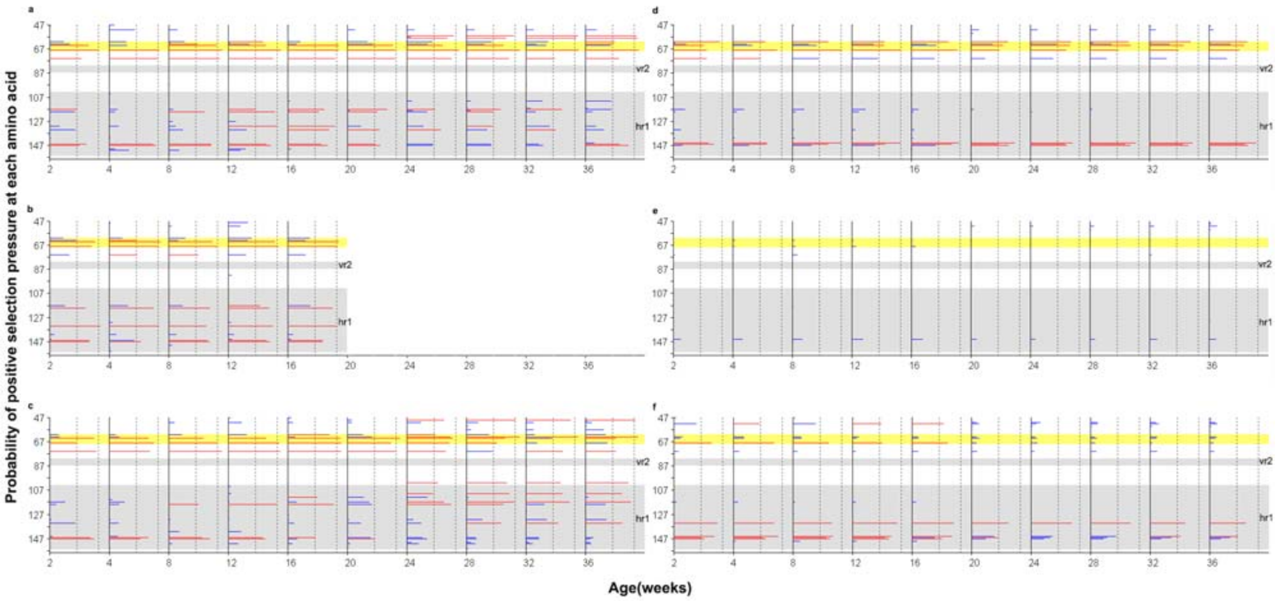

Fig S3. Site-by-site positive selection of the gp85-A gene based on the MEME method. (**A**, **B** and **C**) The site-by-site positive selection value by the MEME method in chickens 1-3, respectively. (**D**, **E** and **F**) The site-by-site positive selection value by the MEME method in chickens 4-6, respectively. The x-axis of each panel represents different sampling time-points. The y-axis represents the amino acid positions of the gp85 protein. The horizontal lines in each panel represent the positively selected sites, and the length of the lines represents the proportion of the identified positively selected sites in the bootstrap re-sampling replicates. Red lines indicate that these amino acid sites are positively selected in at least 50% of the bootstrap re-sampling replicates, and blue lines indicate positive selection of less than 50%. The two vertical dashed lines represent the 50% and 90% thresholds. The two previously described hypervariable regions, vr2 and hr1, are shown using a grey background. Positions 61-68, a novel hypervariable region, are highlighted using a yellow background.

Figure S4

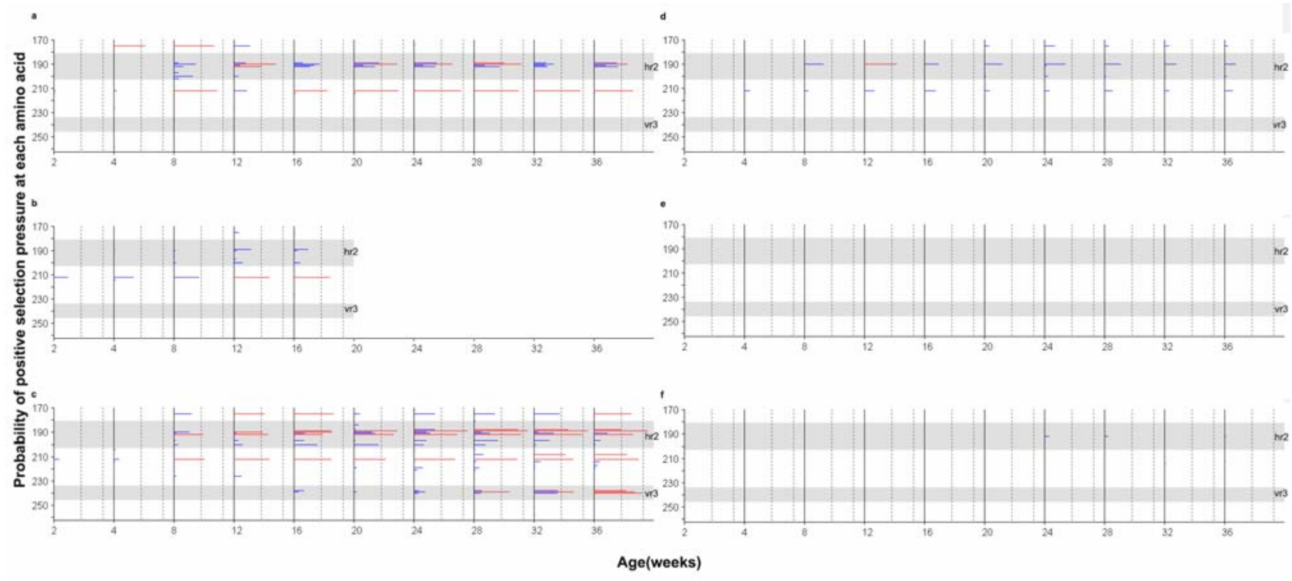

Fig S4. Site-by-site positive selection of the gp85-B gene based on the two rate FEL method. (**A**, **B** and **C**) The site-by-site positive selection value by the two rate FEL method in chickens 1-3, respectively. (**D**, **E** and **F**) The site-by-site positive selection value by the two rate FEL method in chickens 4-6, respectively. The x-axis of each panel represents different sampling time-points. The y-axis represents the amino acid positions of the gp85 protein. The horizontal lines in each panel represent the positively selected sites, and the length of the lines represents the proportion of the identified positively selected sites in the bootstrap re-sampling replicates. Red lines indicate that these amino acid sites are positively selected in at least 50% of the bootstrap re-sampling replicates, and blue lines indicate positive selection of less than 50%. The two vertical dashed lines represent the 50% and 90% thresholds. The two previously described hypervariable regions, vr3 and hr2, are shown using a grey background.

Figure S5

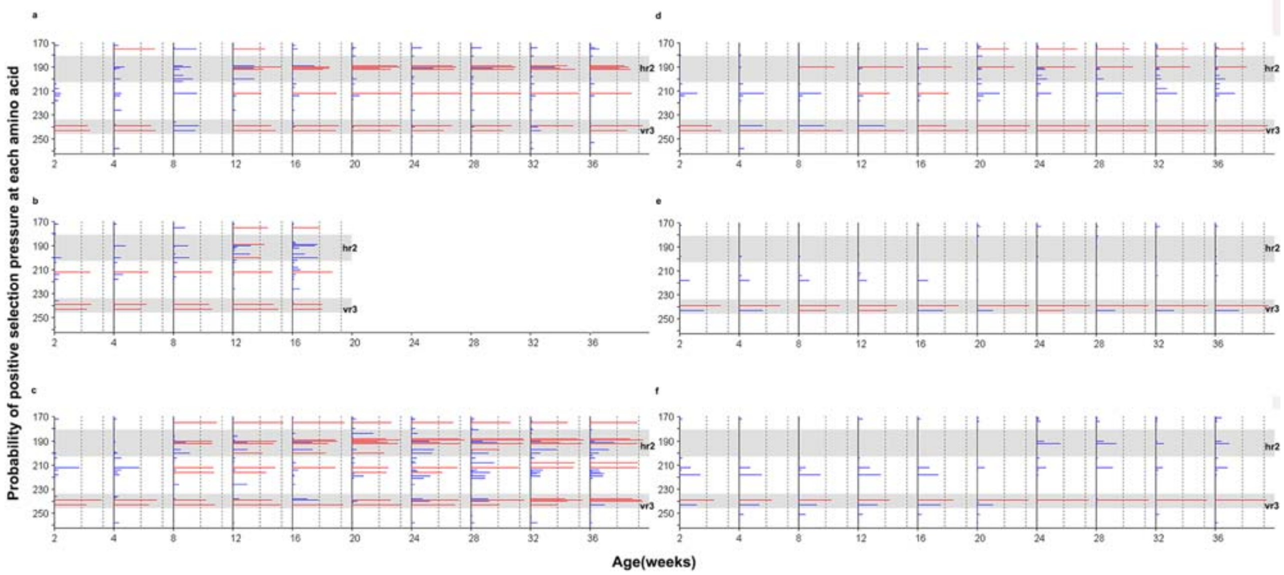

Fig S5. Site-by-site positive selection of the gp85-B gene based on the MEME method.

(**A**, **B** and **C**) The site-by-site positive selection value by the MEME method in chickens 1-3, respectively. (**D**, **E** and **F**) The site-by-site positive selection value by the MEME method in chickens 4-6, respectively. The x-axis of each panel represents different sampling time-points. The y-axis represents the amino acid positions of the gp85 protein. The horizontal lines in each panel represent the positively selected sites, and the length of the lines represents the proportion of the identified positively selected sites in the bootstrap re-sampling replicates. Red lines indicate that these amino acid sites are positively selected in at least 50% of the bootstrap re-sampling replicates, and blue lines indicate positive selection of less than 50%. The two vertical dashed lines represent the 50% and 90% thresholds. The two previously described hypervariable regions, vr3 and hr2, are shown using a grey background.

Table S1. The re-sampling strategy used in the positive selection analysis.

Number	Number of replicates	Number of sequences of each replicate
N≥50000	750	200
40000≤N<50000	600	200
30000≤N<40000	450	200
N<30000	300	200

This table describes the re-sampling strategy used in the positive selection analysis. For example, if the number of clean reads in the dataset is no less than 50000, we performed 750 re-sampling replicates, with 200 sequences in each replicate. If the number of clean reads in the dataset is no less than 40000, but less than 50000, we performed 600 re-sampling replicates, with 200 sequences in each replicate. For each replicate, we performed the positive selection analysis and calculated the global ω and the average ω of all the replicates from a single dataset was estimated to represent the selection level for the dataset.

Table S2. The number of raw reads and clean reads of each sample.

Segment A						Segment B					
Number	Raw reads	Clean reads	Number	Raw reads	Clean reads	Number	Raw reads	Clean reads	Number	Raw reads	Clean reads
A102	46394	45268	A402	57995	56422	B102	44259	43187	B402	28159	27387
A104	38959	37592	A404	44065	42501	B104	44224	42644	B404	50424	48580
A108	52667	44317	A408	21401	20943	B108	24619	23873	B408	34521	33585
A112	34309	33615	A412	7553	7415	B112	48628	47374	B412	50438	49300
A116	16187	15747	A416	22046	21669	B116	43859	42668	B416	29575	28988
A120	37490	34644	A420	28016	26945	B120	17157	16701	B420	7522	7324
A124	32238	31183	A424	47797	46394	B124	17440	17082	B424	31096	30372
A128	32940	31689	A428	29394	28571	B128	12306	12038	B428	19583	19211
A132	46835	45086	A432	24685	23910	B132	11849	11481	B432	10549	10311
A136	25226	23308	A436	41012	39680	B136	7584	7342	B436	17120	16648
A202	42711	41637	A502	15064	14773	B202	40370	39302	B502	18992	18653
A204	10951	10614	A504	44068	42924	B204	36341	35139	B504	47633	46368
A208	36719	36073	A508	14791	14477	B208	37063	36232	B508	31911	31267
A212	3081	3028	A512	38120	37549	B212	48486	47389	B512	48168	47391
A216	33254	32604	A516	38589	37993	B216	41619	40651	B516	38648	38006
			A520	48578	47284				B520	13781	13486
			A524	43742	42877				B524	11856	11662
			A528	24393	23891				B528	8529	8388
			A532	43181	42147				B532	14338	14069
			A536	23005	22173				B536	12329	12073
A302	44164	43092	A602	37799	37055	B302	52977	51698	B602	41360	40420
A304	20522	19921	A604	29273	28781	B304	33163	32031	B604	34250	33629
A308	42011	35350	A608	16051	15781	B308	45770	44615	B608	18006	17671
A312	41748	39956	A612	35969	33077	B312	41373	40410	B612	35631	35038
A316	10735	10534	A616	19509	19213	B316	10988	10767	B616	20914	20545
A320	20369	19422	A620	26176	25427	B320	9027	8755	B620	6287	6150
A324	22155	21222	A624	21189	20686	B324	7034	6794	B624	12401	12162
A328	22436	21525	A628	45032	43819	B328	5212	4991	B628	9949	9772
A332	15692	14936	A632	41373	40109	B332	12203	11617	B632	25171	24629
A336	34528	33016	A636	36206	34758	B336	12845	12264	B636	13323	12893

In this table, the variants is named as follows. For example, A102 means region A of the gp85 gene (gp85-A), in which the first number “1” means chicken 1 and 02 represents that this sample is collected at week 2. Therefore, B424 represents the number of reads of gp85-B at week 24 of chicken 4.

Table S3. List of the positively selected positions of gp85-A estimated using the two rate FEL method in chickens with antibody.

						Week								Week							Week					
	Position	A102	A104	A108	A112	A116	A120	A124	A128	A132	A136	A202	A204	A208	A212	A216	A302	A304	A308	A312	A316	A320	A324	A328	A332	A336
	48	0	1	2	1	2	2	1	1	1	1	0	3	0	38	0	1	0	0	0	2	0	1	2	1	3
	49	0	0	0	0	0	0	0	0	0	1	0	0	0	0	0	0	0	0	0	0	10	69	92	84	93
	50	0	0	0	0	0	0	0	0	0	0	0	0	0	0	0	0	0	0	0	0	0	0	0	1	3
	51	0	49	3	0	0	2	2	6	3	2	0	0	6	24	0	0	1	12	26	10	4	14	5	4	2
	52	0	0	0	0	0	0	0	0	0	0	2	0	1	2	3	0	0	0	0	0	0	0	0	0	0
	53	0	0	0	0	0	0	0	0	0	0	0	0	0	0	0	0	0	0	0	0	0	0	0	0	0
	54	0	0	0	0	0	1	1	0	1	1	0	0	0	0	0	0	0	0	0	0	0	0	2	1	2
	55	0	0	0	0	0	0	0	0	0	0	0	0	0	0	0	0	0	0	0	0	0	1	0	0	2
	56	0	0	0	0	0	1	88	88	98	94	0	0	0	0	0	0	0	0	0	0	0	0	0	0	0
	57	0	0	0	0	0	0	0	0	0	0	0	0	0	0	0	0	0	0	0	0	0	1	10	15	34
	58	0	0	0	0	0	1	76	73	94	97	0	0	0	0	0	0	0	0	0	0	0	0	0	0	0
	59	0	0	0	0	0	0	0	0	0	0	0	0	0	0	0	0	0	0	0	0	0	0	0	0	0
	60	0	0	0	0	0	0	0	0	0	0	0	0	0	0	0	0	0	0	0	0	0	0	0	0	0
	61	26	32	32	64	17	35	47	46	38	51	23	26	29	42	40	15	10	25	34	77	23	28	18	4	13
	62	0	0	1	0	0	0	0	0	0	0	0	0	0	0	0	0	0	0	0	0	0	1	0	0	2
	63	37	1	53	41	12	47	37	30	17	51	50	50	18	38	34	19	19	0	0	15	45	81	99	97	100
	64	73	34	90	71	95	85	67	70	38	46	85	96	82	87	95	82	74	65	71	99	98	86	72	48	58
	65	0	0	0	0	0	0	0	0	0	0	0	0	0	0	0	0	0	0	0	0	0	0	0	0	0
	66	0	0	0	0	0	0	0	0	0	0	0	0	0	0	0	0	0	0	0	0	0	0	0	0	0
	67	0	0	0	0	0	0	0	0	0	0	0	0	0	0	0	0	0	0	0	0	0	0	0	0	0
	68	90	94	100	99	95	90	98	100	100	100	77	92	90	94	93	50	83	92	95	100	82	79	56	21	41
	69	0	0	0	0	0	0	0	0	0	0	0	0	0	0	0	0	0	0	0	0	0	0	0	0	0
	70	0	0	0	0	0	0	0	0	0	0	0	0	0	0	0	0	0	0	0	0	0	0	0	0	0
	71	0	0	0	0	0	0	0	0	0	0	0	0	0	0	0	0	0	0	0	0	0	0	0	0	0
	72	0	0	0	0	0	0	0	0	0	0	0	0	0	0	0	0	0	0	0	0	0	0	0	0	0
	73	0	1	0	0	1	0	0	0	0	1	0	0	0	0	0	0	1	0	0	1	0	0	0	0	0
	74	0	0	0	0	0	0	0	0	0	0	0	0	0	0	0	0	0	0	0	0	0	0	0	0	0
	75	37	84	93	81	82	87	81	74	44	50	31	48	52	28	29	86	73	100	97	99	90	69	47	63	55
	76	0	0	0	0	0	0	0	0	0	0	0	0	0	0	0	0	0	0	0	0	0	0	0	0	0
	77	0	0	0	0	0	0	0	1	0	0	0	0	0	1	0	0	0	0	0	0	0	0	0	0	0
	78	0	0	0	0	0	0	0	0	0	0	0	0	0	0	0	0	0	0	0	0	0	0	0	0	0
	79	0	0	0	0	0	0	0	0	0	0	0	0	0	0	0	0	0	0	0	0	0	0	0	0	0
	80	0	0	0	0	0	0	0	0	0	0	0	0	0	0	0	0	0	0	0	0	0	0	0	0	0
	81	0	0	0	0	0	0	0	0	0	0	0	0	0	0	0	0	0	0	0	0	0	0	0	0	0
	82	0	0	0	0	0	0	0	0	0	0	0	0	0	0	0	0	0	0	0	0	0	0	0	0	0
vr2	83	0	0	0	0	0	0	0	0	0	0	0	0	0	0	0	0	0	0	0	0	0	0	0	0	0
	84	0	0	0	0	0	0	0	0	0	0	0	0	0	0	0	0	0	0	0	0	0	0	0	0	0
	85	0	0	0	0	0	0	0	0	0	0	0	0	0	0	0	0	0	0	0	0	0	0	0	0	0
	86	0	0	0	0	0	0	0	0	0	0	0	0	0	0	0	0	0	0	0	0	0	0	0	0	0
	87	0	0	0	0	0	0	0	0	0	0	0	0	0	0	0	0	0	0	0	0	0	0	0	0	0
	88	0	0	0	0	0	0	0	0	0	0	0	0	0	0	0	0	0	0	0	0	0	0	0	1	0
	89	0	0	0	0	0	0	0	0	0	0	0	0	0	0	0	0	0	0	0	0	0	0	0	0	0
	90	0	0	0	0	0	0	0	0	0	0	0	0	0	0	0	0	0	0	0	0	0	0	0	0	0
	91	0	0	0	0	0	0	0	0	0	0	0	1	0	0	0	0	0	0	0	0	0	0	0	0	0
	92	0	0	0	0	0	0	0	0	0	0	0	0	0	0	0	0	0	0	0	0	0	0	0	0	0
	93	0	0	0	0	0	0	0	0	0	0	0	0	0	0	0	0	0	0	0	0	0	0	0	0	0
	94	0	0	0	0	1	0	0	0	0	0	0	0	0	0	0	0	0	0	0	0	0	0	0	0	0
	95	0	0	0	0	0	0	0	0	0	0	0	0	0	0	0	0	0	0	0	0	0	0	0	0	0
	96	0	0	0	0	0	0	0	0	0	0	0	0	0	0	0	1	2	1	0	0	0	0	1	0	0
	97	1	0	1	0	0	0	0	0	2	1	0	0	0	0	0	0	0	0	0	0	0	0	0	0	0
	98	0	0	0	0	0	0	0	0	0	0	0	0	0	0	0	0	0	0	0	0	0	0	0	0	0
	99	0	0	0	0	0	0	0	0	0	0	0	0	0	0	0	0	0	0	0	0	0	0	0	0	0
	100	0	0	0	0	0	0	0	0	0	0	0	0	0	0	0	0	0	0	0	0	0	0	0	0	0
	101	0	0	0	0	0	0	0	0	0	0	0	0	0	0	0	0	0	0	0	3	0	53	68	57	76
	102	0	0	0	0	0	0	0	0	0	0	0	0	0	0	0	0	0	0	0	0	0	0	0	0	0
	103	0	0	0	0	0	0	0	0	0	0	0	0	0	0	0	0	0	0	0	0	0	0	0	0	0
	104	0	4	0	0	0	0	0	0	0	0	0	0	0	0	0	0	0	1	5	0	0	0	0	0	0
	105	0	0	0	0	0	0	0	0	0	0	0	0	0	0	0	0	0	0	0	0	0	0	0	0	0
	106	0	0	0	0	0	0	0	0	0	0	0	0	0	0	0	0	0	0	0	0	0	0	0	0	0
	107	0	0	0	0	0	0	0	0	0	0	0	0	0	0	0	0	0	0	0	0	0	0	0	0	0
	108	0	0	0	0	0	0	0	0	0	0	0	0	0	0	0	0	0	0	0	0	0	0	0	0	0
	109	0	0	0	0	0	0	0	0	0	0	0	0	0	0	0	0	0	0	0	0	0	0	0	0	0
	110	0	2	0	0	5	2	8	9	31	48	0	0	0	0	0	0	0	0	6	0	0	50	80	69	68
	111	0	0	0	0	0	0	0	0	0	0	0	0	0	0	0	0	0	0	0	0	0	0	0	0	0
	112	0	0	0	0	0	0	0	0	0	0	0	0	0	0	0	0	0	0	0	0	1	0	0	0	0
	113	0	0	0	0	0	0	0	0	0	0	0	0	0	0	0	0	0	0	1	56	31	39	5	1	6
	114	0	0	0	0	0	0	0	0	0	0	0	0	0	0	0	0	0	0	0	0	0	0	0	0	1
	115	0	0	0	0	0	0	0	0	0	0	0	0	0	0	0	0	1	0	0	0	0	1	1	1	0
	116	0	0	0	0	0	0	0	1	9	3	0	0	0	0	0	0	0	0	0	0	0	0	0	0	0
	117	51	16	10	50	69	75	53	64	67	49	29	36	27	59	43	29	30	2	2	17	42	68	89	81	84
	118	0	0	0	0	0	0	0	0	0	0	0	0	0	0	0	0	0	0	0	0	0	0	0	0	0
	119	2	1	30	50	36	40	30	46	16	10	20	35	19	18	31	1	1	2	3	28	25	54	43	18	16
	120	0	0	0	0	1	1	0	0	0	0	0	0	0	0	0	1	0	0	0	0	0	0	0	0	0
	121	0	0	0	0	0	0	0	0	0	0	0	0	0	0	0	0	0	0	0	0	0	0	0	0	0
	122	0	0	0	0	0	0	0	0	0	0	0	0	0	0	0	0	0	0	0	0	0	0	0	0	0
	123	0	0	0	0	0	0	0	0	0	0	0	0	0	0	0	0	0	0	0	0	0	0	0	0	0
	124	0	0	0	0	0	0	0	0	0	0	0	0	0	0	0	0	0	0	0	0	0	0	0	1	0
	125	0	0	0	0	0	0	0	0	0	0	0	0	0	0	0	0	0	0	0	0	0	0	0	0	0
	126	0	0	0	0	0	0	0	0	0	0	0	0	0	0	0	0	0	0	0	0	0	0	0	0	0
	127	1	2	8	14	2	3	0	0	0	0	0	0	0	0	0	0	0	0	0	0	0	0	0	0	0
	128	0	0	0	0	0	0	0	0	0	0	0	0	0	0	0	0	0	0	0	0	0	0	0	0	0
hr1	129	0	0	0	0	0	0	0	0	0	0	0	0	0	0	0	0	0	0	0	0	0	0	0	0	0
	130	0	0	0	0	0	0	0	0	0	0	0	0	0	0	0	0	0	0	0	0	0	0	0	0	0
	131	16	13	5	70	69	13	11	20	35	12	0	6	1	3	1	4	3	3	2	2	3	8	21	32	27
	132	0	0	0	0	0	0	0	0	0	0	0	0	0	0	0	0	0	0	0	0	0	0	0	0	0
	133	0	0	0	0	0	0	0	0	0	0	0	0	0	0	0	0	0	0	0	0	0	0	0	0	0
	134	49	0	27	34	75	58	60	32	52	30	95	93	70	83	90	48	15	2	0	10	23	25	63	55	65
	135	0	0	0	0	0	0	0	0	0	0	0	0	0	0	0	0	0	0	0	0	0	0	0	0	0
	136	0	0	0	0	0	0	0	0	0	0	0	0	0	0	0	0	0	0	0	0	0	0	0	0	0
	137	0	0	0	0	0	0	0	0	0	0	0	0	0	0	0	0	0	0	0	0	0	0	0	0	0
	138	0	0	0	0	0	0	0	0	0	0	0	0	0	0	0	0	0	1	1	0	0	2	1	0	0
	139	0	0	0	0	0	0	0	0	0	0	0	0	0	0	0	0	0	0	0	0	0	0	0	0	0
	140	0	0	0	0	0	0	0	0	0	0	0	0	0	0	0	0	0	0	0	0	0	0	0	0	0
	141	2	3	0	2	2	1	0	1	1	1	10	13	14	13	11	1	1	20	25	1	2	2	0	1	0
	142	0	0	0	0	0	0	0	0	0	0	0	0	0	0	0	0	0	0	0	0	0	0	0	0	0
	143	0	1	1	0	0	0	0	1	0	0	0	0	0	0	0	0	0	0	1	0	1	0	0	0	0
	144	0	0	0	0	0	0	0	0	0	0	0	0	0	0	0	0	0	0	0	0	0	0	0	0	0
	145	0	0	0	0	0	0	0	0	0	6	0	0	0	0	0	1	0	0	0	0	0	0	0	0	0
	146	0	0	0	0	0	0	0	0	0	0	0	0	0	0	0	0	0	0	0	0	0	0	0	0	0
	147	0	0	0	0	1	0	0	0	0	0	0	0	0	0	0	0	0	0	1	0	1	1	0	0	1
	148	0	0	0	0	0	0	0	0	0	0	0	0	0	0	0	0	0	0	0	0	0	0	0	0	0
	149	0	0	0	0	0	0	0	0	0	0	0	0	0	0	0	0	0	0	0	0	0	0	0	0	0
	150	0	11	2	32	6	0	2	2	1	1	0	1	8	2	2	0	0	2	0	3	3	8	9	7	9
	151	0	36	20	24	2	2	2	3	0	0	0	0	2	1	0	0	0	5	17	9	6	22	16	13	8
	152	0	0	0	0	0	0	0	0	0	0	0	0	0	0	0	0	0	0	0	0	0	0	0	0	0
	153	0	0	0	0	0	0	0	0	0	0	0	0	0	0	0	0	0	0	0	0	0	0	0	0	0
	154	0	0	3	0	0	1	0	0	1	0	1	0	0	0	0	0	0	0	0	0	0	0	0	0	1
	155	0	0	0	0	0	0	0	0	1	0	0	1	0	0	0	0	2	0	0	0	1	0	3	3	1

p-values of < 0.05 were considered to be significant. Percentage greater than 50 are highlighted in pink. A102, “A” refers to region A of the gp85 gene (gp85-A), “1” means chicken 1, and “02” represents week 2; therefore, A102 represents the gp85-A of chicken 1 at week 2.

Table S4. List of the positively selected positions of gp85-A estimated using the two rate FEL method in chickens without antibody.

		Week										Week										Week									
	**Position**	**A402**	**A404**	**A408**	**A412**	**A416**	**A420**	**A424**	**A428**	**A432**	**A436**	**A502**	**A504**	**A508**	**A512**	**A516**	**A520**	**A524**	**A528**	**A532**	**A536**	**A602**	**A604**	**A608**	**A612**	**A616**	**A620**	**A624**	**A628**	**A632**	**A636**
	**48**	1	0	2	0	2	2	1	0	2	2	0	0	0	0	0	0	0	0	0	0	0	0	0	0	0	0	0	0	0	1
	**49**	0	0	0	0	0	0	0	0	0	0	0	0	0	0	0	0	0	0	0	0	0	0	0	0	0	0	0	0	0	0
	**50**	0	0	0	0	0	0	0	0	0	0	0	0	0	0	0	0	0	0	0	0	0	0	0	0	0	2	1	1	0	1
	**51**	0	0	0	0	0	1	1	1	1	0	0	0	0	0	0	0	0	0	0	0	0	0	0	0	0	0	0	0	0	0
	**52**	0	0	0	0	0	0	0	0	0	0	0	0	0	0	0	0	0	0	0	0	44	50	42	54	59	14	10	14	17	15
	**53**	0	0	0	0	0	0	0	0	0	0	0	0	0	0	0	0	0	0	0	0	0	0	0	0	0	0	0	0	0	0
	**54**	0	0	0	0	0	3	0	1	0	1	0	0	0	0	0	1	0	1	2	2	0	0	0	0	0	1	1	1	2	2
	**55**	0	0	0	0	0	0	0	0	0	0	0	0	0	0	0	0	0	0	0	0	0	0	0	0	0	0	0	0	0	0
	**56**	0	0	0	0	0	0	0	0	0	0	0	0	0	0	0	0	0	0	0	0	0	0	0	0	0	0	0	0	0	0
	**57**	0	0	0	0	0	0	0	0	0	0	0	0	0	0	0	0	0	0	0	0	0	0	0	0	0	0	0	0	0	0
	**58**	0	0	0	0	0	0	0	0	0	0	0	0	0	0	0	0	0	0	0	0	0	0	0	0	0	0	0	0	0	0
	**59**	0	0	0	0	0	0	0	0	0	0	0	0	0	0	0	0	0	0	0	0	0	0	0	0	0	0	0	0	0	0
	**60**	0	0	0	0	0	0	0	0	0	0	0	0	0	0	0	0	0	0	0	0	0	0	0	0	0	0	0	0	0	0
	**61**	87	59	64	58	66	64	74	69	71	68	0	0	0	0	0	0	0	0	0	0	0	0	0	0	0	0	0	0	0	0
	**62**	0	0	0	0	0	0	0	0	0	0	0	0	0	0	0	0	0	0	0	0	0	0	0	0	0	0	0	0	1	0
	**63**	24	28	32	41	30	42	49	39	38	41	2	5	5	4	3	2	1	1	3	1	17	21	16	13	12	9	9	8	13	13
	**64**	57	37	46	60	47	61	74	76	64	66	0	0	1	0	0	0	1	2	0	1	13	21	17	8	13	16	11	13	13	11
	**65**	0	0	0	0	0	0	0	0	0	0	0	0	0	0	0	0	0	0	0	0	0	0	0	0	0	0	0	0	0	0
	**66**	0	0	0	0	0	0	0	0	0	0	0	0	0	0	0	0	0	0	0	0	0	0	0	0	0	0	0	0	0	0
	**67**	0	0	0	0	0	0	0	0	0	0	0	0	0	0	0	0	0	0	0	0	0	0	0	0	0	0	0	0	0	0
	**68**	62	83	93	90	87	54	66	53	55	56	2	4	6	8	9	1	0	0	0	0	70	75	67	55	67	9	11	9	5	9
	**69**	0	0	0	0	0	0	0	0	0	0	0	0	0	0	0	0	0	0	0	0	0	0	0	0	0	0	0	0	0	0
	**70**	0	0	0	0	0	0	0	0	0	0	0	0	0	0	0	0	0	0	0	0	0	0	0	0	0	0	0	0	0	0
	**71**	0	0	0	0	0	0	0	0	0	0	0	0	0	0	0	0	0	0	0	0	0	0	0	0	0	0	0	0	0	0
	**72**	0	0	0	0	0	0	0	0	0	0	0	0	0	0	0	0	0	0	0	0	0	0	0	0	0	0	0	0	0	0
	**73**	1	1	1	0	0	0	0	0	0	0	0	0	0	0	0	0	0	0	0	0	0	0	0	0	0	0	1	0	0	0
	**74**	0	0	0	0	0	0	0	0	0	0	0	0	0	0	0	0	0	0	0	0	0	0	0	0	0	0	0	0	0	0
	**75**	60	49	45	41	39	22	38	29	26	27	1	1	3	2	1	0	0	0	1	1	8	9	7	4	9	8	5	8	9	7
	**76**	0	0	0	0	0	0	0	0	0	0	0	0	0	0	0	0	0	0	0	0	0	0	0	0	0	0	0	0	0	0
	**77**	0	0	0	0	0	0	0	0	0	0	0	0	0	0	0	0	0	0	0	0	0	0	0	0	0	0	0	0	0	0
	**78**	0	0	0	0	0	0	0	0	0	0	0	0	0	0	0	0	0	0	0	0	0	0	0	0	0	0	0	0	0	0
	**79**	0	0	0	0	0	0	0	0	0	0	0	0	0	0	0	0	0	0	0	0	0	0	0	0	0	0	0	0	0	0
	**80**	0	0	0	0	0	0	0	0	0	0	0	0	0	0	0	0	0	0	0	0	0	0	0	0	0	0	0	0	0	0
	**81**	0	0	0	0	0	0	0	0	0	0	0	0	0	0	0	0	0	0	0	0	0	0	0	0	0	0	0	0	0	0
	**82**	0	0	0	0	0	0	0	0	0	0	0	0	0	0	0	0	0	0	0	0	0	0	0	0	0	0	0	0	0	0
**vr2**	**83**	0	0	0	0	0	0	0	0	0	0	0	0	0	0	0	0	0	0	0	0	0	0	0	0	0	0	0	0	0	0
	**84**	0	0	0	0	0	0	0	0	0	0	0	0	0	0	0	0	0	0	0	0	0	0	0	0	0	0	0	0	0	0
	**85**	0	0	0	0	0	0	0	0	0	0	0	0	0	0	0	0	0	0	0	0	0	0	0	0	0	0	0	0	0	0
	**86**	0	0	0	0	0	0	0	0	0	0	0	0	0	0	0	0	0	0	0	0	0	0	0	0	0	0	0	0	0	0
	**87**	0	0	0	0	0	0	0	0	0	0	0	0	0	0	0	0	0	0	0	0	0	0	0	0	0	0	0	0	0	0
	**88**	0	0	0	0	0	0	0	0	0	0	0	0	0	0	0	0	0	0	0	0	0	0	0	0	0	0	0	0	0	0
	**89**	0	0	0	0	0	0	0	0	0	0	0	0	0	0	0	0	0	0	0	0	0	0	0	0	0	0	0	0	0	0
	**90**	0	0	0	0	0	0	0	0	0	0	0	0	0	0	0	0	0	0	0	0	0	0	0	0	0	0	0	0	0	0
	**91**	0	0	0	0	0	0	0	0	0	0	0	0	0	0	0	0	0	0	0	0	0	0	0	0	0	0	0	0	0	0
	**92**	0	0	0	0	0	0	0	0	0	0	0	0	0	0	0	0	0	0	0	0	0	0	0	0	0	0	0	0	0	0
	**93**	0	0	0	0	0	0	0	0	0	0	0	0	0	0	0	0	0	0	0	0	0	0	0	0	0	0	0	0	0	0
	**94**	0	0	0	0	0	0	0	0	0	0	0	0	0	0	0	0	0	0	0	0	0	0	0	0	0	0	0	0	0	0
	**95**	0	0	0	0	0	0	0	0	0	0	0	0	0	0	0	0	0	0	0	0	0	0	0	0	0	0	0	0	0	0
	**96**	0	0	0	0	0	0	0	0	0	0	0	0	0	0	0	0	0	0	0	0	0	0	0	0	0	0	0	0	0	0
	**97**	0	0	0	0	0	0	0	0	0	0	0	0	0	0	0	0	0	0	0	0	0	0	0	0	0	0	0	0	0	0
	**98**	0	0	0	0	0	0	0	0	0	0	0	0	0	0	0	0	0	0	0	0	0	0	0	0	0	0	0	0	0	0
	**99**	0	0	0	0	0	0	0	0	0	0	0	0	0	0	0	0	0	0	0	0	0	0	0	0	0	0	0	0	0	0
	**100**	0	0	0	0	0	0	0	0	0	0	0	0	0	0	0	0	0	0	0	0	0	0	0	0	0	0	0	0	0	0
	**101**	0	0	0	0	0	0	0	0	0	0	0	0	0	0	0	0	0	0	0	0	0	0	0	0	0	0	0	0	0	0
	**102**	0	0	0	0	0	0	0	0	0	0	0	0	0	0	0	0	0	0	0	0	0	0	0	0	0	0	0	0	0	0
	**103**	0	0	0	0	0	0	0	0	0	0	0	0	0	0	0	0	0	0	0	0	0	0	0	0	0	0	0	0	0	0
	**104**	0	0	0	0	0	0	0	0	0	0	0	0	0	0	0	0	0	0	0	0	0	0	0	0	0	0	0	0	0	0
	**105**	0	0	0	0	0	0	0	0	0	0	0	0	0	0	0	0	0	0	0	0	0	0	0	0	0	0	0	0	0	0
	**106**	0	0	0	0	0	0	0	0	0	0	0	0	0	0	0	0	0	0	0	0	0	0	0	0	0	0	0	0	0	0
	**107**	0	0	0	0	0	0	0	0	0	0	0	0	0	0	0	0	0	0	0	0	0	0	0	0	0	0	0	0	0	0
	**108**	0	0	0	0	0	0	0	0	0	0	0	0	0	0	0	0	0	0	0	0	0	0	0	0	0	0	0	0	0	0
	**109**	0	1	0	0	0	0	0	0	0	0	0	0	0	0	0	0	0	0	0	0	0	0	0	0	0	0	0	0	0	0
	**110**	0	0	0	0	0	0	0	0	0	0	0	0	0	0	0	0	0	0	0	0	0	0	0	0	0	0	0	0	0	0
	**111**	0	0	0	0	0	0	0	0	0	0	0	0	0	0	0	0	0	0	0	0	0	0	0	0	0	0	0	0	0	0
	**112**	0	0	0	0	0	0	0	0	0	0	0	0	0	0	0	0	0	0	0	0	0	0	0	0	0	0	0	0	0	0
	**113**	0	0	0	0	0	0	0	0	0	0	0	0	0	0	0	0	0	0	0	0	0	0	0	0	0	0	1	0	0	0
	**114**	0	0	0	0	0	0	0	0	0	0	0	0	0	0	0	0	0	0	0	0	0	0	0	0	0	0	0	0	0	0
	**115**	0	1	0	0	0	0	0	0	0	0	0	0	0	0	0	0	0	0	0	0	0	0	0	0	0	0	0	0	0	0
	**116**	0	0	0	0	0	0	0	0	0	0	0	0	0	0	0	0	0	0	0	0	0	0	0	0	0	0	0	0	0	0
	**117**	23	22	22	26	14	6	1	5	2	1	1	0	2	0	0	0	0	0	1	1	6	10	5	7	8	1	2	2	1	2
	**118**	0	0	0	0	0	0	0	0	0	0	0	0	0	0	0	0	0	0	0	0	0	0	0	0	0	0	0	0	0	0
	**119**	0	0	0	0	0	0	0	0	0	0	0	0	0	0	0	0	0	0	0	0	0	0	0	0	0	0	0	0	0	0
	**120**	0	0	1	0	0	0	0	0	0	0	0	0	0	0	0	0	0	0	0	0	0	0	0	0	0	0	0	0	0	0
	**121**	0	0	0	0	0	0	0	0	0	0	0	0	0	0	0	0	0	0	0	0	0	0	0	0	0	0	0	0	0	0
	**122**	0	0	0	0	0	0	0	0	0	0	0	0	0	0	0	0	0	0	0	0	0	0	0	0	0	0	0	0	0	0
	**123**	0	0	0	0	0	0	0	0	0	0	0	0	0	0	0	0	0	0	0	0	0	0	0	0	0	0	0	0	0	0
	**124**	0	0	0	0	0	0	0	0	0	0	0	0	0	0	0	0	0	0	0	0	0	0	0	0	0	0	0	0	0	0
	**125**	0	0	0	0	0	0	0	0	0	0	0	0	0	0	0	0	0	0	0	0	0	0	0	0	0	0	0	0	0	0
	**126**	0	0	0	0	0	0	0	0	0	0	0	0	0	0	0	0	0	0	0	0	0	0	0	0	0	0	0	0	0	0
	**127**	0	0	0	0	0	0	0	0	0	0	0	0	0	0	0	0	0	0	0	0	0	0	0	0	0	0	0	0	0	0
	**128**	0	0	0	0	0	0	0	0	0	0	0	0	0	0	0	0	0	0	0	0	0	0	0	0	0	0	0	0	0	0
**hr1**	**129**	0	0	0	0	0	0	0	0	0	0	0	0	0	0	0	0	0	0	0	0	0	0	0	0	0	0	0	0	0	0
	**130**	0	0	0	0	0	0	0	0	0	0	0	0	0	0	0	0	0	0	0	0	0	0	0	0	0	0	0	0	0	0
	**131**	0	0	0	0	0	0	0	0	0	0	0	0	0	0	0	0	0	0	0	0	0	0	0	0	0	0	0	0	0	0
	**132**	0	0	0	0	0	0	0	0	0	0	0	0	0	0	0	0	0	0	0	0	0	0	0	0	0	0	0	0	0	0
	**133**	0	0	0	0	0	0	0	0	0	0	0	0	0	0	0	0	0	0	0	0	0	0	0	0	0	0	0	0	0	0
	**134**	15	3	4	8	6	0	0	0	0	0	0	0	0	0	0	0	0	0	0	0	81	74	72	84	77	70	76	74	65	68
	**135**	0	0	0	0	0	0	0	0	0	0	0	0	0	0	0	0	0	0	0	0	0	0	0	0	0	0	0	0	0	0
	**136**	0	0	0	0	0	0	0	0	0	0	0	0	0	0	0	0	0	0	0	0	0	0	0	0	0	0	0	0	0	0
	**137**	0	0	0	0	0	0	0	0	0	0	0	0	0	0	0	0	0	0	0	0	0	0	0	0	0	0	0	0	0	0
	**138**	0	0	0	0	0	0	0	0	0	0	0	0	0	0	0	0	0	0	0	0	0	0	1	0	0	0	0	0	0	0
	**139**	0	0	0	0	0	0	0	0	0	0	0	0	0	0	0	0	0	0	0	0	0	0	0	0	0	0	0	0	0	0
	**140**	0	0	0	0	0	0	0	0	0	0	0	0	0	0	0	0	0	0	0	0	0	0	0	0	0	0	0	0	0	0
	**141**	4	4	5	2	3	1	1	0	0	0	0	0	0	0	0	0	0	0	0	0	1	1	2	1	0	1	1	1	1	1
	**142**	0	0	0	0	0	0	0	0	0	0	0	0	0	0	0	0	0	0	0	0	0	0	0	0	0	0	0	0	0	0
	**143**	0	0	0	0	0	0	1	0	0	0	0	0	0	0	0	0	0	0	0	0	0	0	0	0	0	0	0	0	0	0
	**144**	0	0	0	0	0	0	0	0	0	0	0	0	0	0	0	0	0	0	0	0	0	0	0	0	0	0	0	0	0	0
	**145**	10	16	43	38	52	53	48	53	52	65	1	3	3	3	3	5	7	4	8	10	18	16	12	17	19	16	20	22	24	24
	**146**	0	0	0	0	0	0	0	0	0	0	0	0	0	0	0	0	0	0	0	0	0	0	0	0	0	0	0	0	0	0
	**147**	0	0	0	0	0	0	0	0	0	0	0	0	0	0	0	0	0	0	0	0	0	0	0	0	0	0	0	0	0	0
	**148**	0	0	0	0	0	0	0	0	0	0	0	0	0	0	0	0	0	0	0	0	0	0	0	0	0	0	0	0	0	0
	**149**	0	0	0	0	0	0	0	0	0	0	0	0	0	0	0	0	0	0	0	0	0	0	0	0	0	0	0	0	0	0
	**150**	0	0	0	1	2	2	2	1	2	4	0	0	0	0	0	0	0	0	0	0	0	0	0	0	0	0	0	0	0	0
	**151**	0	0	0	0	1	1	1	1	1	2	0	0	0	0	0	0	0	0	0	0	0	0	0	0	0	0	0	0	0	0
	**152**	0	0	0	0	0	0	0	0	0	0	0	0	0	0	0	0	0	0	0	0	0	0	0	0	0	0	0	0	0	0
	**153**	0	0	0	0	0	0	0	0	0	0	0	0	0	0	0	0	0	0	0	0	0	0	0	0	0	0	0	0	0	0
	**154**	0	0	0	0	0	0	0	0	0	0	0	0	0	0	0	0	0	0	0	0	0	0	0	0	0	0	0	0	0	1
	**155**	0	1	0	0	0	0	0	0	0	0	0	0	0	0	0	0	0	0	0	0	0	0	0	0	0	0	0	0	0	1

p-values of < 0.05 were considered to be significant. Percentage greater than 50 are highlighted in pink. A402, “A” refers to region A of the gp85 gene (gp85-A), “4” means chicken 4, and “02” represents week 2; therefore, A402 represents the gp85-A of chicken 4 at week 2.

Table S5. List of the positively selected positions of gp85-B estimated using the two rate FEL method in chickens with antibody.

		Week										Week							Week							
	Position	B102	B104	B108	B112	B116	B120	B124	B128	B132	B136	B202	B204	B208	B212	B216	B302	B304	B308	B312	B316	B320	B324	B328	B332	B336
	170	0	0	0	0	0	0	0	0	0	0	0	0	0	0	0	0	0	0	0	0	0	0	0	0	0
	171	0	0	0	0	0	0	0	0	0	0	0	0	0	0	0	0	0	0	0	1	0	0	0	0	0
	172	1	2	0	1	1	0	0	0	0	1	2	1	0	1	0	2	0	1	0	0	2	3	0	1	0
	173	0	0	0	0	0	0	0	0	0	0	0	0	0	0	0	0	0	0	0	0	0	0	0	0	0
	174	0	0	1	0	0	1	2	1	1	0	0	0	0	0	0	0	0	0	0	0	0	0	0	0	0
	175	0	59	75	31	1	0	0	0	1	2	0	0	1	11	3	0	0	34	58	74	12	40	40	48	69
	176	0	0	0	0	0	0	0	0	0	0	0	0	0	0	0	0	0	0	0	0	0	0	0	0	0
	177	0	0	0	0	0	0	0	0	0	0	0	0	0	0	0	0	0	0	0	0	0	0	0	0	0
	178	0	0	0	0	0	0	0	0	0	0	0	0	0	0	0	0	0	0	0	0	0	0	0	0	0
	179	0	0	0	0	0	0	0	0	0	0	0	0	0	0	0	0	0	0	0	0	0	0	0	0	0
	180	1	0	0	0	0	0	0	0	0	0	1	0	0	0	0	1	1	0	0	0	0	0	1	0	0
	181	0	0	1	0	0	1	0	1	0	1	0	0	0	0	0	0	0	0	0	0	0	1	4	2	2
	182	0	0	0	0	0	0	0	0	0	0	0	0	0	0	0	0	0	0	0	0	0	0	0	0	0
	183	0	0	0	0	0	0	0	0	0	0	0	0	0	0	0	0	0	0	0	0	0	0	0	0	0
	184	0	0	0	0	0	0	0	0	0	0	0	0	0	0	0	0	0	0	0	1	9	0	0	0	0
	185	0	0	0	0	0	0	0	0	0	0	0	0	0	0	0	0	0	0	0	0	0	0	0	0	0
	186	0	0	0	0	0	0	0	0	0	0	0	0	0	0	0	0	0	0	1	0	0	0	0	0	0
	187	0	0	0	0	0	0	0	0	0	0	0	0	0	0	0	0	0	0	0	0	0	0	0	0	0
	188	0	0	0	0	0	0	0	0	0	0	0	0	0	0	0	0	0	0	0	0	4	40	81	64	51
	189	0	0	10	23	17	47	43	57	22	43	0	0	0	33	27	0	0	0	0	68	80	100	100	100	99
hr2	190	0	1	42	78	48	81	71	87	37	62	0	3	4	6	7	0	0	29	54	71	33	24	15	9	2
	191	0	1	1	13	38	19	18	21	22	23	0	0	0	1	0	0	0	5	5	20	40	31	30	37	26
	192	0	0	18	50	31	40	40	49	26	45	0	0	0	0	2	0	0	55	64	54	74	80	88	89	73
	193	0	0	0	0	0	0	0	0	0	0	0	0	0	0	0	0	0	0	0	0	0	0	0	0	0
	194	0	0	0	0	0	0	0	0	0	0	0	0	0	0	0	0	0	0	0	0	0	0	0	0	0
	195	0	0	0	0	0	0	0	0	0	0	0	0	0	0	0	0	0	0	0	0	0	0	0	0	0
	196	0	0	0	0	0	0	0	0	0	0	0	0	0	0	0	0	0	0	0	0	0	0	0	0	0
	197	0	0	10	0	0	0	0	0	0	0	0	0	0	5	2	0	0	4	9	19	4	24	45	29	13
	198	0	0	0	0	0	0	2	0	1	0	0	0	0	0	0	0	0	0	0	0	0	0	0	1	0
	199	0	0	0	0	0	0	0	0	0	0	0	0	0	0	0	0	0	0	0	0	0	0	0	0	0
	200	0	1	37	10	1	2	2	0	0	1	0	1	5	17	13	0	1	8	17	44	47	20	22	5	4
	201	0	0	0	0	0	0	0	0	0	0	0	0	0	0	0	0	0	0	0	0	0	0	0	0	0
	202	0	0	10	0	0	0	0	0	0	0	0	0	0	1	1	0	0	0	0	0	0	0	0	0	0
	203	0	0	0	0	0	0	0	0	0	0	0	0	0	0	0	0	0	0	0	0	0	0	0	0	0
	204	0	1	0	1	1	1	1	1	1	2	1	1	1	1	1	1	4	2	0	0	1	2	2	1	1
	205	0	0	0	0	0	0	0	0	0	0	0	0	0	0	0	0	0	0	0	0	0	0	0	0	0
	206	0	0	0	0	0	0	0	0	0	0	0	0	0	0	0	0	0	0	0	0	0	0	0	0	0
	207	0	0	0	0	1	0	1	1	0	0	0	0	0	0	0	0	0	0	0	0	0	0	0	0	0
	208	0	0	0	0	0	0	0	0	0	0	0	0	0	0	2	0	0	0	0	1	0	0	18	58	62
	209	0	0	0	0	0	0	0	0	0	0	0	0	0	0	0	0	0	0	0	0	0	0	0	0	0
	210	0	0	0	0	0	0	0	0	0	0	0	0	0	0	2	0	0	0	0	0	0	0	0	0	0
	211	0	0	0	0	0	0	0	0	0	0	0	0	0	0	0	0	0	0	0	0	0	0	0	0	0
	212	4	6	80	25	63	83	87	83	86	73	27	38	47	66	68	11	11	57	67	70	59	76	81	73	83
	213	0	0	0	0	0	0	0	0	0	0	0	0	0	0	0	0	0	0	0	0	0	0	0	0	0
	214	3	2	1	1	4	4	0	0	2	3	2	4	0	1	1	1	2	1	1	2	0	3	5	13	12
	215	0	0	0	0	0	0	0	0	0	0	0	0	0	0	0	0	0	0	0	0	0	0	0	0	1
	216	0	0	0	0	0	0	0	0	0	0	0	0	0	0	0	0	0	0	0	0	0	1	0	0	0
	217	0	0	0	0	0	0	0	1	1	0	0	0	0	0	0	0	0	0	0	0	0	0	0	3	8
	218	0	0	0	0	0	0	0	0	0	0	0	0	0	0	0	0	0	0	0	0	0	0	0	0	0
	219	0	0	0	0	0	0	0	0	0	0	0	0	0	0	0	0	0	0	0	1	6	17	11	3	4
	220	0	0	0	0	0	0	0	0	0	0	0	0	0	0	0	0	0	0	0	0	0	0	0	0	0
	221	0	0	0	0	0	0	0	0	0	0	0	0	0	0	0	0	0	0	0	0	1	7	4	0	0
	222	0	0	0	0	0	0	0	0	0	0	0	0	0	0	0	0	0	0	0	0	0	0	0	0	0
	223	0	0	0	0	0	0	0	0	0	0	0	0	0	0	0	0	0	0	0	0	0	0	0	0	0
	224	0	0	0	0	0	0	0	0	0	0	0	0	0	0	0	0	0	0	0	0	0	0	0	0	0
	225	0	0	0	0	0	0	0	0	0	0	0	0	0	0	0	0	0	0	0	0	0	0	0	0	0
	226	0	3	0	3	1	0	0	1	0	0	0	0	0	2	3	0	0	5	15	0	0	0	0	0	0
	227	0	0	0	0	0	0	0	0	0	0	0	0	0	0	0	0	0	0	0	0	0	0	0	0	0
	228	0	0	0	0	0	0	0	0	0	0	0	0	0	0	0	0	0	0	0	0	0	0	0	0	0
	229	0	0	0	0	0	0	0	0	0	0	0	0	0	0	0	0	0	0	0	0	0	0	0	0	0
	230	0	0	0	0	0	0	0	0	0	0	0	0	0	0	0	0	0	0	0	0	0	0	0	0	0
	231	0	0	0	0	0	0	0	0	0	0	0	0	0	0	0	0	0	0	0	0	0	0	0	0	0
	232	0	0	0	0	0	0	0	0	0	0	0	0	0	0	0	0	0	0	0	0	0	0	0	0	0
	233	0	0	0	0	0	0	0	0	0	0	0	0	0	0	0	0	0	0	0	0	0	0	0	0	0
	234	0	0	0	0	0	0	0	0	0	0	0	0	0	0	0	0	0	0	0	0	0	0	0	0	0
	235	0	0	0	0	0	0	0	0	0	0	0	0	0	0	0	0	0	0	0	0	0	0	0	0	0
	236	0	0	3	0	1	0	0	0	0	0	0	0	1	0	0	0	0	0	0	0	0	0	0	0	0
	237	0	0	0	0	0	0	0	0	0	0	0	0	0	0	0	0	1	0	0	0	0	0	0	0	0
vr3	238	0	0	0	0	0	0	0	0	0	0	0	0	0	0	1	0	0	0	2	19	1	10	16	45	60
	239	0	0	0	0	0	0	0	0	0	0	0	0	0	0	1	0	0	2	1	9	6	21	66	74	77
	240	0	0	0	0	1	0	0	0	2	0	0	0	0	0	0	0	0	0	0	0	2	10	13	44	90
	241	0	0	0	0	0	0	0	0	0	0	0	0	0	0	0	0	0	0	0	0	0	0	0	0	0
	242	0	0	0	0	0	0	0	0	0	0	0	0	0	0	0	0	0	0	0	0	0	0	0	0	0
	243	0	0	0	0	0	0	0	0	0	0	0	0	0	0	0	0	0	0	0	0	0	0	0	0	0
	244	0	0	0	0	0	0	0	0	0	0	0	0	0	0	0	0	0	0	1	0	0	0	0	0	0
	245	0	0	0	0	0	0	0	0	0	0	0	0	0	0	0	0	0	0	0	0	0	0	0	0	0
	246	0	0	0	0	0	0	0	0	0	0	0	0	0	0	0	0	0	0	0	0	0	0	0	0	0
	247	0	0	0	0	0	0	0	0	0	0	0	0	0	0	0	0	0	0	0	0	0	0	0	0	0
	248	0	0	0	0	0	0	0	0	0	0	0	0	0	0	0	0	0	0	0	0	0	0	0	0	0
	249	0	0	0	0	0	0	0	0	0	0	0	0	0	0	0	0	0	0	0	0	0	0	0	0	0
	250	0	0	0	0	0	0	0	0	0	0	0	0	0	0	0	0	0	0	0	0	0	0	0	0	0
	251	0	0	0	0	0	0	0	0	0	0	0	0	0	0	0	0	0	0	0	0	0	0	0	0	0
	252	0	0	0	0	0	0	0	0	0	0	0	0	0	0	0	0	0	0	0	0	0	0	0	0	0
	253	0	0	0	0	0	0	0	0	0	0	0	0	0	0	0	0	0	0	0	0	0	0	1	0	0
	254	0	0	0	0	0	0	0	0	0	0	0	0	0	0	0	0	0	0	0	0	0	0	0	0	0
	255	0	0	0	0	0	0	0	0	0	0	0	0	0	0	0	0	0	0	0	0	0	0	0	0	0
	256	0	0	0	0	0	0	0	0	0	0	0	0	0	0	0	0	0	0	0	0	0	0	0	0	0
	257	0	0	0	0	0	0	0	0	0	0	0	0	0	0	0	0	0	0	0	0	0	0	0	0	0
	258	0	3	0	0	0	0	0	0	0	0	0	0	0	0	0	0	1	0	1	0	0	0	1	2	1
	259	0	0	0	0	0	0	0	0	0	0	0	0	0	0	0	0	0	0	0	0	0	0	0	0	0

p-values of < 0.05 were considered to be significant. Percentage greater than 50 are highlighted in pink. B102, “B” refers to region B of the gp85 gene (gp85-B), “1” means chicken 1, and “02” represents week 2; therefore, B102 represents the gp85-B of chicken 1 at week 2.

Table S6. List of the positively selected positions of gp85-B estimated using the two rate FEL method in chickens without antibody.

		Week										Week										Week									
	Position	B402	B404	B408	B412	B416	B420	B424	B428	B432	B436	B502	B504	B508	B512	B516	B520	B524	B528	B532	B536	B602	B604	B608	B612	B616	B620	B624	B628	B632	B636
	170	0	0	0	0	0	0	0	0	0	0	0	0	0	0	0	0	0	0	0	0	0	0	0	0	0	0	0	0	0	0
	171	0	0	0	0	0	0	0	0	0	0	0	0	0	0	0	0	0	0	0	0	0	0	0	0	0	0	0	0	0	0
	172	0	1	0	0	0	1	0	1	0	0	1	0	0	0	0	0	0	0	0	1	1	0	0	1	1	0	0	0	0	2
	173	0	0	0	0	0	0	0	0	0	0	0	0	0	0	0	0	0	0	0	0	0	0	0	0	0	0	0	0	0	0
	174	0	0	0	0	0	0	0	0	0	0	0	0	0	0	0	0	0	0	0	0	0	0	0	0	0	0	0	0	0	0
	175	0	0	0	0	0	9	20	9	9	7	0	0	0	0	0	0	0	0	0	0	0	0	0	0	0	0	0	0	0	0
	176	0	0	0	0	0	0	0	0	0	0	0	0	0	0	0	0	0	0	0	0	0	0	0	0	0	0	0	0	0	0
	177	0	0	0	0	0	0	0	0	0	0	0	0	0	0	0	0	0	0	0	0	0	0	0	0	0	0	0	0	0	0
	178	0	0	0	0	0	0	0	0	0	0	0	0	0	0	0	0	0	0	0	0	0	0	0	0	0	0	0	0	0	0
	179	0	0	0	0	0	0	0	0	0	0	0	0	0	0	0	0	0	0	0	0	0	0	0	0	0	0	0	0	0	0
	180	0	1	0	0	0	0	0	0	0	0	0	0	0	0	0	0	0	0	0	0	0	0	0	0	0	0	0	0	0	0
	181	0	0	0	0	0	1	0	0	1	1	0	0	0	0	0	1	0	0	1	0	0	0	0	0	0	1	0	0	0	0
	182	0	0	0	0	0	0	0	0	0	0	0	0	0	0	0	0	0	0	0	0	0	0	0	0	0	0	0	0	0	0
	183	0	0	0	0	0	0	0	0	0	0	0	0	0	0	0	0	0	0	0	0	0	0	0	0	0	0	0	0	0	0
	184	0	0	0	0	0	0	0	0	0	0	0	0	0	0	0	0	0	0	0	0	0	0	0	0	0	0	0	0	0	0
	185	0	0	0	0	0	0	0	0	0	0	0	0	0	0	0	0	0	0	0	0	0	0	0	0	0	0	0	0	0	0
	186	0	0	0	0	0	0	0	0	0	0	0	0	0	0	0	0	0	0	0	0	0	0	0	0	0	0	0	0	0	0
	187	0	0	0	0	0	0	0	0	0	0	0	0	0	0	0	0	0	0	0	0	0	0	0	0	0	0	0	0	0	0
	188	0	0	0	0	0	0	0	0	0	0	0	0	0	0	0	0	0	0	0	0	0	0	0	0	0	0	0	0	0	0
	189	0	0	0	0	0	0	0	0	0	0	0	0	0	0	0	0	0	0	0	0	0	0	0	0	0	0	0	0	0	0
hr2	190	0	0	36	61	27	34	40	31	23	21	0	0	0	0	0	0	0	0	1	0	0	0	0	0	0	0	2	2	0	1
	191	0	0	0	1	1	1	4	1	0	1	0	0	0	0	0	0	0	0	0	0	0	0	0	0	0	0	0	0	0	0
	192	0	0	0	1	0	1	3	1	0	0	0	0	0	0	0	0	0	0	0	0	0	0	0	0	0	0	9	7	1	4
	193	0	0	0	0	0	0	0	0	0	0	0	0	0	0	0	0	0	0	0	0	0	0	0	0	0	0	0	0	0	0
	194	0	0	0	0	0	0	0	0	0	0	0	0	0	0	0	0	0	0	0	0	0	0	0	0	0	0	0	0	0	0
	195	0	0	0	0	0	0	0	0	0	0	0	0	0	0	0	0	0	0	0	0	0	0	0	0	0	0	0	0	0	0
	196	0	0	0	0	0	0	0	0	0	0	0	0	0	0	0	0	0	0	0	0	0	0	0	0	0	0	0	0	0	0
	197	0	0	0	0	0	0	1	0	0	0	0	0	0	0	0	0	0	0	0	0	0	0	0	0	0	0	0	0	0	0
	198	0	0	0	0	0	1	0	0	0	1	0	1	0	0	0	0	0	0	0	0	0	0	0	0	0	0	0	0	0	0
	199	0	0	0	0	0	0	0	0	0	0	0	0	0	0	0	0	0	0	0	0	0	0	0	0	0	0	0	0	0	0
	200	0	0	0	0	0	4	7	4	3	5	0	0	0	0	0	0	0	0	0	0	0	0	0	0	0	0	0	0	0	0
	201	0	0	0	0	0	0	0	0	0	0	0	0	0	0	0	0	0	0	0	0	0	0	0	0	0	0	0	0	0	0
	202	0	0	0	0	0	0	0	0	0	1	0	0	0	0	0	0	0	0	0	0	0	0	0	0	0	0	0	0	0	0
	203	0	0	0	0	0	0	0	0	0	0	0	0	0	0	0	0	0	0	0	0	0	0	0	0	0	0	0	0	0	0
	204	1	2	1	0	1	2	1	1	0	0	0	1	0	0	0	1	0	0	0	0	0	0	0	0	0	0	0	0	1	1
	205	0	0	0	0	0	0	0	0	0	0	0	0	0	0	0	0	0	0	0	0	0	0	0	0	0	0	0	0	0	0
	206	0	0	0	0	0	0	0	0	0	0	0	0	0	0	0	0	0	0	0	0	0	0	0	0	0	0	0	0	0	0
	207	0	0	0	0	0	0	0	0	0	0	0	0	0	0	0	0	0	0	0	0	0	0	0	0	0	0	0	0	0	0
	208	0	0	0	0	0	0	0	0	0	0	0	0	0	0	0	0	0	0	0	0	0	0	0	0	0	0	0	0	0	0
	209	0	0	0	0	0	0	0	0	0	0	0	0	0	0	0	0	0	0	0	0	0	0	0	0	0	0	0	0	0	0
	210	0	0	0	0	0	0	0	0	0	0	0	0	0	0	0	0	0	0	0	0	0	0	0	0	0	0	0	0	0	0
	211	0	0	0	0	0	0	0	0	0	0	0	0	0	0	0	0	0	0	0	0	0	0	0	0	0	0	0	0	0	0
	212	3	11	9	19	22	11	10	16	10	16	0	0	0	0	0	0	0	0	0	0	2	2	1	1	1	0	2	2	1	3
	213	0	0	0	0	0	0	0	0	0	0	0	0	0	0	0	0	0	0	0	0	0	0	0	0	0	0	0	0	0	0
	214	3	1	0	1	1	2	0	0	0	2	0	0	1	0	0	0	0	1	0	1	0	0	2	0	0	0	1	0	2	1
	215	0	0	0	0	0	0	0	0	0	0	0	0	0	0	0	0	0	0	0	0	0	0	0	0	0	0	0	0	0	0
	216	0	0	0	0	0	0	0	0	0	0	0	0	0	0	0	0	0	0	0	0	0	0	0	0	0	0	0	0	0	0
	217	1	1	0	0	0	0	0	0	0	0	0	0	0	0	0	0	0	0	0	0	0	0	0	0	0	0	0	0	0	0
	218	0	0	0	0	0	0	0	0	0	0	0	0	0	0	0	0	0	0	0	0	0	0	0	0	0	0	0	0	0	0
	219	0	0	0	0	0	0	0	0	0	0	0	0	0	0	0	0	0	0	0	0	0	0	0	0	0	0	0	0	0	0
	220	0	0	0	0	0	0	0	0	0	0	0	0	0	0	0	0	0	0	0	0	0	0	0	0	0	0	0	0	0	0
	221	0	0	0	0	0	0	0	0	0	0	0	0	0	0	0	0	0	0	0	0	0	0	0	0	0	0	0	0	0	0
	222	0	0	0	0	0	0	0	0	0	0	0	0	0	0	0	0	0	0	0	0	0	0	0	0	0	0	0	0	0	0
	223	0	0	0	0	0	0	0	0	0	0	0	0	0	0	0	0	0	0	0	0	0	0	0	0	0	0	0	0	0	0
	224	0	0	0	0	0	0	0	0	0	0	0	0	0	0	0	0	0	0	0	0	0	0	0	0	0	0	0	0	0	0
	225	0	0	0	0	0	0	0	0	0	0	0	0	0	0	0	0	0	0	0	0	0	0	0	0	0	0	0	0	0	0
	226	0	0	0	0	0	0	0	0	0	0	0	0	0	0	0	0	0	0	0	0	0	0	0	0	0	0	0	0	0	0
	227	0	0	0	0	0	0	0	0	0	0	0	0	0	0	0	0	0	0	0	0	0	0	0	0	0	0	0	0	0	0
	228	0	0	0	0	0	0	0	0	0	0	0	0	0	0	0	0	0	0	0	0	0	0	0	0	0	0	0	0	0	0
	229	0	0	0	0	0	0	0	0	0	0	0	0	0	0	0	0	0	0	0	0	0	0	0	0	0	0	0	0	0	0
	230	0	0	0	0	0	0	0	0	0	0	0	0	0	0	0	0	0	0	0	0	0	0	0	0	0	0	0	0	0	0
	231	0	0	0	0	0	0	0	0	0	0	0	0	0	0	0	0	0	0	0	0	0	0	0	0	0	0	0	0	0	0
	232	0	0	0	0	0	0	0	0	0	0	0	0	0	0	0	0	0	0	0	0	0	0	0	0	0	0	0	0	0	0
	233	0	0	0	0	0	0	0	0	0	0	0	0	0	0	0	0	0	0	0	0	0	0	0	0	0	0	0	0	0	0
	234	0	0	0	0	0	0	0	0	0	0	0	0	0	0	0	0	0	0	0	0	0	0	0	0	0	0	0	0	0	0
	235	0	0	0	0	0	0	0	0	0	0	0	0	0	0	0	0	0	0	0	0	0	0	0	0	0	0	0	0	0	0
	236	0	0	0	0	0	0	0	0	0	0	0	0	0	0	0	0	0	0	0	0	0	0	0	0	0	0	0	0	0	0
	237	0	0	0	0	0	0	0	0	0	0	0	0	0	0	0	0	0	0	0	0	0	0	0	0	0	0	0	0	0	0
vr3	238	0	0	0	0	0	0	0	0	0	0	0	0	0	0	0	0	0	0	0	0	0	0	0	0	0	0	0	0	0	0
	239	0	0	0	0	0	0	0	0	0	0	0	0	0	0	0	0	0	0	0	0	0	0	0	0	0	0	0	0	0	0
	240	0	0	0	0	0	0	0	0	0	0	0	0	0	0	0	0	0	0	0	0	0	0	0	0	0	0	0	0	0	0
	241	0	0	0	0	0	0	0	0	0	0	0	0	0	0	0	0	0	0	0	0	0	0	0	0	0	0	0	0	0	0
	242	0	0	0	0	0	0	0	0	0	0	0	0	0	0	0	0	0	0	0	0	0	0	0	0	0	0	0	0	0	0
	243	0	0	0	0	0	0	0	0	0	0	0	0	0	0	0	0	0	0	0	0	0	0	0	0	0	0	0	0	0	0
	244	0	0	0	0	0	0	0	0	0	0	0	0	0	0	0	0	0	0	0	0	0	0	0	0	0	0	0	0	0	0
	245	0	0	0	0	0	0	0	0	0	0	0	0	0	0	0	0	0	0	0	0	0	0	0	0	0	0	0	0	0	0
	246	0	0	0	0	0	0	0	0	0	0	0	0	0	0	0	0	0	0	0	0	0	0	0	0	0	0	0	0	0	0
	247	0	0	0	0	0	0	0	0	0	0	0	0	0	0	0	0	0	0	0	0	0	0	0	0	0	0	0	0	0	0
	248	0	0	0	0	0	0	0	0	0	0	0	0	0	0	0	0	0	0	0	0	0	0	0	0	0	0	0	0	0	0
	249	0	0	0	0	0	0	0	0	0	0	0	0	0	0	0	0	0	0	0	0	0	0	0	0	0	0	0	0	0	0
	250	0	0	0	0	0	0	0	0	0	0	0	0	0	0	0	0	0	0	0	0	0	0	0	0	0	0	0	0	0	0
	251	0	0	0	0	0	0	0	0	0	0	0	0	0	0	0	0	0	0	0	0	0	0	0	0	0	0	0	0	0	0
	252	0	0	0	0	0	0	0	0	0	0	0	0	0	0	0	0	0	0	0	0	0	0	0	0	0	0	0	0	0	0
	253	0	0	0	0	0	0	0	0	0	0	0	0	0	0	0	0	0	0	0	0	0	0	0	0	0	0	0	0	0	0
	254	0	0	0	0	0	0	0	0	0	0	0	0	0	0	0	0	0	0	0	0	0	0	0	0	0	0	0	0	0	0
	255	0	0	0	0	0	0	0	0	0	0	0	0	0	0	0	0	0	0	0	0	0	0	0	0	0	0	0	0	0	0
	256	0	0	0	0	0	0	0	0	0	0	0	0	0	0	0	0	0	0	0	0	0	0	0	0	0	0	0	0	0	0
	257	0	0	0	0	0	0	0	0	0	0	0	0	0	0	0	0	0	0	0	0	0	0	0	0	0	0	0	0	0	0
	258	0	1	0	0	0	0	0	0	0	0	0	0	0	0	0	0	0	0	0	0	0	0	0	0	0	0	0	0	0	1
	259	0	0	0	0	0	0	0	0	0	0	0	0	0	0	0	0	0	0	0	0	0	0	0	0	0	0	0	0	0	0

p-values of < 0.05 were considered to be significant.Percentage greater than 50 are highlighted in pink. B402, “B” refers to region B of the gp85 gene (gp85-B), “4” means chicken 4, and “02” represents week 2; therefore, B402 represents the gp85-B of chicken 4 at week 2.
